# Possible Role for Allelic Variation in Yeast *MED15* in Ecological Adaptation

**DOI:** 10.3389/fmicb.2021.741572

**Published:** 2021-10-18

**Authors:** David G. Cooper, Yishuo Jiang, Sydney Skuodas, Luying Wang, Jan S. Fassler

**Affiliations:** Biology Department, University of Iowa, Iowa City, IA, United States

**Keywords:** *MED15*/*GAL11*, wine yeast, domestication, Mediator, polyglutamine tracts, fermentation, ethanol

## Abstract

The propensity for *Saccharomyces cerevisiae* yeast to ferment sugars into ethanol and CO_2_ has long been useful in the production of a wide range of food and drink. In the production of alcoholic beverages, the yeast strain selected for fermentation is crucial because not all strains are equally proficient in tolerating fermentation stresses. One potential mechanism by which domesticated yeast may have adapted to fermentation stresses is through changes in the expression of stress response genes. *MED15* is a general transcriptional regulator and RNA Pol II Mediator complex subunit which modulates the expression of many metabolic and stress response genes. In this study, we explore the role of *MED15* in alcoholic fermentation. In addition, we ask whether *MED15* alleles from wine, sake or palm wine yeast improve fermentation activity and grape juice fermentation stress responses. And last, we investigate to what extent any differences in activity are due to allelic differences in the lengths of three polyglutamine tracts in *MED15*. We find that strains lacking *MED15* are deficient in fermentation and fermentation stress responses and that *MED15* alleles from alcoholic beverage yeast strains can improve both the fermentation capacity and the response to ethanol stresses when transplanted into a standard laboratory strain. Finally, we find that polyglutamine tract length in the Med15 protein is one determinant in the efficiency of the alcoholic fermentation process. These data lead to a working model in which polyglutamine tract length and other types of variability within transcriptional hubs like the Mediator subunit, Med15, may contribute to a reservoir of transcriptional profiles that may provide a fitness benefit in the face of environmental fluctuations.

## Introduction

*MED15* encodes a subunit of the tail domain of the RNA Polymerase II Mediator complex. Mediator is a conserved transcriptional regulatory complex consisting of 21 core subunits in yeast (Guglielmi et al., 2004), divided into three domains (head, middle, and tail) that helps recruit RNA Pol II to target genes (Davis et al., 2002). The primary function of the head and middle modules is to interact with RNA Pol II and the general transcriptional machinery, while the primary function of the tail module is to interact with DNA-bound transcription factors and chromatin remodeling complex proteins (Myers et al., 1999; Takagi et al., 2006; Takagi and Kornberg, 2006; Ansari et al., 2012; Ansari and Morse, 2012). As a member of the Mediator tail domain Med15 has both positive and negative roles in gene expression (Suzuki et al., 1988; Fassler and Winston, 1989; Sussel et al., 1995; Cooper and Fassler, 2019) that are the result of physical contacts with a variety of transcription factors, chromatin remodeling proteins and other subunits of the Mediator complex. Med15 is required for normal activation of stress response genes and metabolic genes among others, as well as for the downregulation of genes involved in translation and ribosome biogenesis (Cooper and Fassler, 2019). Including both positive and negatively regulated genes, *MED15* regulates as much as 10% of the genome (Ansari et al., 2012). Deletions of the *MED15* gene are viable, but have a reduced growth rate and exhibit sensitivity to many stresses including heat (Auesukaree et al., 2009), low pH (Mollapour et al., 2004), hyperoxia (Outten et al., 2005), and osmotic stress (Zapater et al., 2007). The null mutant also exhibits reduced growth on non-glucose carbon sources (Nogi and Fukasawa, 1980; Suzuki et al., 1988), reduced growth under anaerobic conditions (Samanfar et al., 2013), and reduced respiratory growth rate (Merz and Westermann, 2009; Bode et al., 2013).

The Med15 protein can be subdivided into three large structural domains. The N-terminal ∼90 amino acids comprise a well-conserved and fully characterized KIX (kinase inducible domain interacting domain) (Parker et al., 1996; Thakur et al., 2008). The Med15 KIX domain engages pleiotropic drug resistance transcription factor (Pdr1) orthologs, which are key regulators of the multidrug resistance pathway in *Saccharomyces cerevisiae* and the clinically important human pathogen *Candida glabrata* (Thakur et al., 2008). The C-terminal 250 amino acids, sometimes referred to as the Mediator Association Domain (MAD), is known for its role in Mediator assembly. Deletions throughout this region disrupt Mediator integrity. Residues located between amino acids (aa) 500 and 630 and outside of the MAD domain may also contribute to mediator assembly (Jedidi et al., 2010). The middle domain of Med15 is intrinsically disordered, due in part to the overrepresentation of glutamine residues. The amino acid bias is extensive and the *S. cerevisiae* protein and many fungal Med15 orthologs are characterized by long tracts of uninterrupted glutamine. Fungal Med15 proteins contain three prominent glutamine stretches (here called Q1, Q2, and Q3) which lie adjacent to and partially overlap with the positions of predicted coiled-coil domains (Cooper and Fassler, 2019).

The role for polyglutamine (poly-Q) tracts in Med15 has not been established, though the functional importance of poly-Q has been investigated in other contexts. Poly-Q tract containing proteins are enriched in transcriptional regulators (Cooper and Fassler, 2019) and more specifically there is an enrichment of glutamine residues within transcriptional activation domains (Gerber et al., 1994). Polyglutamine tracts likely effect transcriptional activation by mediating protein-protein interactions as has been observed in Taf12 - Rap1 interactions (Garbett et al., 2007), the homodimerization of Nab3 (Loya et al., 2013), the interaction between Q rich human TBP and TFIIB (Friedman et al., 2007), the interaction between the glutamine-rich activation domain in *Drosophila* SP1 with itself (Pascal and Tjian, 1991) and with Taf4 (Hoey et al., 1993), and the *in vitro* poly-Q-dependent interaction between basal transcription factors such as TFIID and TFIIF with the mutant (expanded Q) Huntingtin (Htt) protein (Zhai et al., 2005).

The availability of thousands of genomes for individual *S. cerevisiae* strains provides an opportunity to evaluate the potential for variation within the Med15 protein. We find occasional amino acid substitutions, but the most striking variation within Med15 is the length of simple sequence repeats that underlie the glutamine and glutamine-alanine tracts in the central part of the protein. Recently, the phenotypic stress response consequences of Med15 polyglutamine variation have been investigated (Gallagher et al., 2020). The significance of this type of variation has also been investigated in the yeast Ssn6 (Cyc8) transcriptional co-repressor protein (Gemayel et al., 2015) as well as in the ANGUSTIFOLIA and Clock proteins in other organisms (O’Malley et al., 2010; Bryan et al., 2018).

The role of *MED15* in industrial phenotypes has not been thoroughly investigated. In one study *MED15* was found to be required for *FLO11* activation and the hydrophobicity phenotype that leads to the flotation of sherry yeast (known as flor) during the aging process in barrels (Barrales et al., 2008; Fidalgo et al., 2008). These and other studies found that *MED15* was also required for flocculation and adhesion (Barrales et al., 2008; Fidalgo et al., 2008). Finally, overexpression of *Arabidopsis thaliana MED15* in wild type strains of *S. cerevisiae* led to increased flocculation, adhesion and a significant increase in ethanol production (Dahiya et al., 2016).

Yeast have been used to ferment grape juice/must in the production of alcoholic beverages for millennia. The differences in wine and beer fermentation environments have resulted in yeast diversification with certain strains becoming adapted to the production of specific beverages (Legras et al., 2018). Genome scale analyses of hundreds of strains have shown that yeast adapt to new environments *via* both small and large genetic changes (Peter et al., 2018). Small variations due to single nucleotide or small indels can change the structure and/or function of the encoded protein or alter gene expression. Larger changes including chromosomal rearrangements, segmental duplications and gene copy number variation have also been observed. Changes in glutamine tract length, which are expected to occur at higher frequencies than the average spontaneous mutation rate (1.7 e-7) (Gou et al., 2019), may also provide a reservoir of standing diversity that could be tapped during the process of adaptation to novel environments.

Grape juice is a stressful environment for yeast due in part to its high sugar content (140–260 g/L total sugar) (14–26°Bx) which is an equimolar mixture of glucose and fructose, resulting from the cleavage of sucrose within the grape (Bisson, 1993). The elevated sugar content in grapes constitutes a severe hyperosmotic stress which is compounded by other constituents of the grape must including soil- and agrochemical-based copper (Provenzano et al., 2010; Sun et al., 2016) as well as the sulfites added immediately after grape crush to inhibit or kill spoilage organisms (Divol et al., 2012). In addition, low concentrations of available nitrogen in grape juice (Henschke and Jiranek, 1993) can become growth limiting, as amino acids are quickly depleted during fermentation (Jiranek et al., 1995). Grape juice also has a low pH (2.8–3.8) caused by a mix of tartaric, malic, and citric acids (Pretorius, 2000). Available nitrogen and ∼50% of the sugars are quickly consumed during the growth phase (Bisson, 1993) of the yeast inoculum. The remaining sugar is fermented after culture saturation in the production of energy. Ethanol is the main byproduct of yeast fermenting glucose for energy. Other metabolic products are made throughout the fermentation process including various acids (acetic, succinic, and lactic) and volatile compounds (esters, fatty acids, aldehydes, and higher alcohols) (Pretorius, 2000; Varela et al., 2012; Peltier et al., 2018).

In addition to the environmental stresses present at the start of fermentation, accumulation of both ethanol and acetic acid are stressors specific to late fermentation cultures. Correspondingly, wine yeasts have high tolerance to many compounds including acetic acid, copper, sulfite, high glucose, and ethanol (Gallone et al., 2016). Yeast respond to the initial and accumulating stresses of grape juice and fermentation through the environmental stress response (Mendes-Ferreira et al., 2007a,b). Ethanol toxicity is thought to occur by disrupting membrane structural integrity, while the addition of lipids or oxygenation that is required to synthesize fatty acids can mitigate the toxic effects (Pretorius, 2000; Varela et al., 2012).

In the present study, we assess the contribution of *MED15* to industrial phenotypes such as growth in high sugar media, ethanol and acetic acid tolerance, ethanol production and fermentation rates. We also investigate the potential contribution of sequence variation within the Med15 protein to the fermentation lifestyle. To investigate this set of questions we constructed isogenic laboratory strains (S288C) that either contain or lack the *MED15* gene (WT and *med15*Δ), as well as *med15*Δ strains carrying centromeric plasmids expressing either the laboratory (LAB) or alleles of *MED15* from strains specific to different types of alcoholic beverage production. All *MED15* genes were expressed from the *MED15* promoter from the genome of the S288C laboratory strain. Commercial strains with polyglutamine tract lengths differing from the LAB strain (Cooper and Fassler, 2019) were used to investigate the impact of natural poly-Q tract allelic variation on the activity of Med15. We find that strains lacking *MED15* have reduced tolerance for growth on ethanol and display reduced fermentation rates. We also find that certain *MED15* alleles from industrial strains improve growth and fermentation rates over strains with in *MED15*_*LAB*_ alleles. The *MED15* sequence of LAB and industrial strains differ primarily, but not exclusively, in the length of their glutamine tracts. Strain reconstruction experiments show that glutamine tract lengths in Med15 do influence fermentation rate, and have the potential to be adaptive in certain domestication niches.

## Materials and Methods

### Strains

Strains are derivatives of S288C, a widely used non-flocculent laboratory strain, originally designed for biochemical studies (Mortimer and Johnston, 1986). The *med15Δ* strain was from the deletion collection (Brachmann et al., 1998). Genotypes of additional strains used in this study are listed in Table 1. Industrial yeast strains were purchased from commercial vendors (WY1, Lalvin 71B-1122, Home Brew Stuff; WY2, Lalvin RC-212 and WY3, Lalvin ICV_D-47, Amazon; WY4, Lalvin EC-1118, Home Brew Ohio; WY5, Lalvin K1-V-1116, RiteBrew) or were obtained from the ARS culture collection^1^ (SY20, sake yeast, NRRL-Y11573 and PY23, palm wine yeast, NRRL-Y17772) or were from the Goddard laboratory collection (Bradbury et al., 2006) (WY7, Lalvin ICV D-254; WY15, DSM Fermichamp).

**TABLE 1:** Strains Used in this Study^1^.

Strain	Relevant genotype	Parental strain and/or plasmids	Source or references
BY4742	MATα *his3Δ1 leu2Δ0 lys2Δ0 ura3Δ0*	(OY235)	Brachmann et al., 1998
(OY320)	*med15Δ0*	BY4742 deletion collection	Brachmann et al., 1998
JF2692	BY4742 *MED15*	pJF2141 in OY320	This study
JF2641	WY7 *MED15*	pYJ2155 in OY320	This study
JF2642	WY15 *MED15*	pYJ2155 in OY320	This study
JF2643	WY20 *MED15*	pYJ2157 in OY320	This study
JF2646	WY23 *MED15*	pYJ2158 in OY320	This study
JF2648	WY1-A *MED15*	pYJ2166 in OY320	This study
JF2649	WY1-B *MED15*	pYJ2167 in OY320	This study
JF2650	WY1-C *MED15*	pYJ2168 in OY320	This study
JF2651	WY2 *MED15*	pYJ2169 in OY320	This study
JF2653	WY3 *MED15*	pYJ2171 in OY320	This study
JF2654	WY4 *MED15*	pYJ2172 in OY320	This study
JF2655	WY5 *MED15*	pYJ2173 in OY320	This study
JF2739	“Clean” WY7 *MED15*	pDC2214 in OY320	This study
JF2740	“Clean” WY15 *MED15*	pDC2209 in OY320	This study
JF2741	“Clean” SY20 *MED15*	pDC2210 in OY320	This study
JF2742	“Clean” PY23 *MED15*	pDC2212 in OY320	This study
WY1	Diploid wine yeast		Lalvin 71B-1122
WY2	Diploid wine yeast		Lalvin RC-212 Bourgovin
WY3	Diploid wine yeast		Lalvin ICV D-47
WY4	Diploid wine yeast		Lalvin EC 1118
WY5	Diploid wine yeast		Lalvin K1-V1116
WY7	Diploid wine yeast		Lalvin ICV D254
WY15	Diploid wine yeast		DSM Fermichamp
SY20	Diploid wine yeast		Kyokai 7
PY23	Diploid wine yeast		NRRL Y-17772

*^1^Parenthetical names (OY###) are lab-specific storage designations.*

### Plasmid Construction

The details of plasmid construction are found in Supplementary File 2. Plasmid used in this work are provided in Supplementary Table 1.

### Primers

Primers used in this work are provided in Supplementary Table 2.

### Yeast Methods

#### Colony PCR

Rapid PCR screening was conducted using Taq polymerase, colony PCR buffer [final concentrations: 12.5 mM Tris-Cl (pH 8.5), 56 mM KCl, 1.5 mM MgCl_2_, 0.2 mM dNTPs], and primers (0.2 μM). A small amount of yeast cells were transferred using the end of a 200 μL micropipet tip. A standard hot-start thermocycler program with extension at 68°C was adjusted for the Tm of the primer set and size of the amplification product.

#### Yeast Transformation

Transformations of *med15*Δ strains were conducted using the frozen-EZ yeast transformation II (Zymo Research) with minor modifications. Transformations into all other strains were conducted using a standard lithium acetate transformation protocol (Ito et al., 1983; Gietz and Woods, 2002).

#### Media

Growth and fermentation rates were compared using various types of media, including rich YPD media where D is glucose/dextrose added to 2% (2%) or 20% (20%) with or without supplements at the indicated concentrations (ethanol, acetic acid); YP media with galactose (2%, YPGalactose) or glycerol (3%, YPGlycerol); or amino-acid supplemented white or red grape juice media (20–21°Bx with added leucine, histidine, lysine, and uracil; (white grape juice, WGJ-AA; red grape juice, RGJ-AA) at the indicated temperatures (°C). Grape juice concentrate (Global Vinters Inc., Canada) supplied at ∼68°Bx (1°Bx = 1 g/100 mL) was diluted to 20–21°Bx with distilled deionized water using a triple scale hydrometer and confirmed with a refractometer (Milwaukee). Grape juice media was supplemented with 200 mg/L histidine, 300 mg/L leucine, 300 mg/L lysine, and 200 mg/L uracil for use in growth and fermentation experiments. The yeast assimilable nitrogen (YAN) in this media (242.5 mg N/L) was determined using the formol titration method (Gump et al., 2001). In some experiments, ammonium sulfate was added to the media at 1 g/L to increase the YAN to (434 mg N/L).

#### Growth Curves

Saturated precultures were prepared in triplicate by overnight incubation in selective media with aeration at 30°C. Cultures were then diluted in the same media and incubated to exponential phase and then diluted to an initial concentration of 5 × 10^6^ cells/mL based on hemocytometer counts. Growth was monitored spectrophotometrically at 600 nm over time. Growth rates and other parameters such as lag and doubling times were analyzed using GrowthRates v3.0 (Hall et al., 2014; Mira et al., 2017; Hall et al., 2020) as an unbiased way to identify datapoints comprising the linear part of the growth curve. Differences among the WY *MED15* alleles the LAB allele and the deletion (*med15*Δ) were evaluated using ANOVA with Tukey *post-hoc* analysis (Tukey, 1949). ^∗^, *p* < 0.05; ^∗∗^, *p* < 0.01; ^∗∗∗^, *p* < 0.001.

#### Spot Assays

Exponentially growing subcultures in selective media were differentially diluted to achieve a consistent initial concentration of 5 × 10^6^ cell/mL. 10-fold serial dilutions were carried out in the wells of a sterile 96 well microtiter dish. 2 μl volumes of each dilution were spotted on different types of solid media. Phenotypes were observed and imaged daily.

### Fermentation Methods

#### Fermentation Reactions

Small scale fermentation reactions were fashioned after Peltier et al. (2018). 5 mL fermentations were conducted in 10 mL molded sterile clear glass vials with tight rubber cap closures (American Clinical Supplies Inc.). The cap was punctured with a 25 G × 7/8 (0.5 mm × 22 mm) hypodermic needle which remained in place to allow CO_2_ release from the system while still restricting evaporation (Peltier et al., 2018). Yeast cultures were prepared for fermentation by incubating in SC-leucine media which selects for the presence of the *MED15* expression plasmid for 2.5 days. After saturation, cultures were diluted 1:20 into fresh SC-leucine media, the cell count determined using a hemocytometer, and the OD_600_ determined spectrophotometrically. 1.25 × 10^7^ cells from saturated cultures were added to 5 mL prepared white grape must with added amino acids (or other media as indicated in the figure legends) in the fermentation vials to a final cell concentration of 2.5 × 10^6^ cell/mL. Uninoculated vials with the same volume of grape must were used to control for contamination. Fermentation vials were incubated at 25 ± 1°C using a heated shaker in a 4°C cold room with agitation at 175 ± 5 rpm. Progression through fermentation was determined using daily weight loss measurements with multiple different transformants (biological replicates) per genotype. In pre-treatment experiments cultures were prepared as above, subcultured and grown to early log phase before being subjected to 38°C or to WGJ (diluted 1:3 in YPD) for 1 h. After treatment, cells were gently pelleted and resuspended in full-strength WGJ in fermentation vials.

#### Ethanol Measurements

Ethanol measurements were averaged over 6 biological replicates. The assay was conducted in punctured fermentation vials identical to those used for weight loss. Samples were evaluated with the K-OH Ethanol Kit (Megazyme) which measures NADH produced following enzymatic conversion of ethanol to acetaldehyde and then to acetic acid and NADH. The Megazyme protocol was adapted for use in microtiter dishes. 10 μL samples consisting of yeast in WGJ were collected daily from vials prepared identical to and in parallel with fermentation vials, diluted in 90 μL of water, and incubated for 1 h at 30°C to release ethanol into the supernatant. 10 μL of each supernatant was further diluted and stored in microtubes until assayed. Reagents (minus the alcohol dehydrogenase enzyme) were combined and added to microtiter dish wells containing the appropriate supernatant dilutions (typically 1:200 for day 1 and 1:1000 for subsequent days) and a background absorbance (A_340_) reading taken. The alcohol dehydrogenase enzyme was then added to each well to initiate the reaction. Following a 5-min incubation at room temperature, spectrophotometric readings were taken every minute until the A_340_ reading stabilized. An ethanol standard at 0.05 μg/μL was included in every experiment. The change in absorbance was used to calculate the concentration of ethanol in g/L using the formula provided.

#### Cell Counts and Viability

To determine cell number and viability in the fermentation cultures, fermentation experiments were set up in duplicate with one vial being evaluated for weight loss and never opened, and a second punctured fermentation vial identical to those used for weight loss sampled once daily throughout the time course. 20 μL samples were removed from each culture for hemocytometer counts and OD_600_ measurements using a plate reader. Diluted samples were also resuspended in 100 μL 1× PBS solution and 100 μL methylene blue (0.1 mg/mL methylene blue in 2% dihydrate sodium citrate) incubated for 5-min and then examined microscopically to estimate the percentage of metabolically inactive (staining blue) cells (*n* = 200 cells).

Viability/vitality was also assessed using the FUN-1 stain {[2-chloro-4-(2,3-dihydro-3-methyl-(benzo-1,3-thiazol-2-yl)-methylidene)-1-phenylquinolinium iodide], Molecular Probes, F7030} according to manufacturer’s directions. Yeast cells were washed in staining buffer (2% glucose and 10 mM Na-HEPES at pH 7.2), resuspended in staining buffer with 2 μM FUN-1, and incubated for 60 min at 30°C in the dark prior to microscopy. In live cells, FUN-1 is converted from a diffusely distributed pool of intracellular green fluorescence to compact orange-red intravacuolar structures, a process that requires both plasma membrane integrity and metabolic capability. Metabolically inactive cells exhibit a uniform green glow and lack fluorescent intravacuolar bodies due to the non-specific distribution of the dye. Extremely bright, diffuse, green-yellow cytoplasmic fluorescence was taken to represent cells that have become permeabilized with consequent rapid uptake of the dye with staining of protein and DNA (Millard et al., 1997). The ratio of cells exhibiting red fluorescence over those exhibiting green or bright yellow fluorescence (*N* > 200 cells) for each strain and time point was used in calculating the percent viable or vital cells in each population.

#### Reactive Oxygen Measurements

Reactive oxygen species levels were measured daily during the fermentation time course using the oxidant-sensitive cell permeable 2’7’ dichlorofluorescein diacetate (H_2_DCFDA) fluorogenic probe (Sigma). Samples were removed from punctured fermentation vials (set up in parallel and not used for weight loss measurements) and processed as previously described (Sinha and Pick, 2021). Fluorescence was measured in 96 well microtiter dishes with a Cytation 5 plate reader. Fluorescence values were calculated by subtracting the fluorescence in unstained samples from the fluorescence in the corresponding stained sample, and normalizing to the culture density (OD_600_).

### Reproducibility and Statistics

Fermentation experiments were conducted using multiple transformants (up to 10) of each plasmid type as indicated in each figure legend. Each experiment was repeated on at least two separate occasions. Fermentation data is plotted using GraphPad Prism 8.4 either as line graphs, or by day using box and whisker plots, with boxes above and below the median representing the 1st and 3rd quartile and whiskers represent the 10th and 90th percentile. Differences between each WY *MED15* allele and the LAB allele or between the *med15*Δ and the LAB allele were evaluated by two-tailed *t*-test, with the Holm-Sidak multiple testing correction (*P*_*adj*_) where indicated, or one-way ANOVA with Tukey (1949) *post-hoc* analysis using GraphPad Prism 8.4 as indicated in the legends ^∗^, *p* 0.05; ^∗∗^, *p* 0.01; ^∗∗∗^, *p* 0.001.

## Results

### *MED15* Is Required for Tolerance to Fermentation Stresses and for Normal Fermentation Kinetics

**FIGURE 1:**
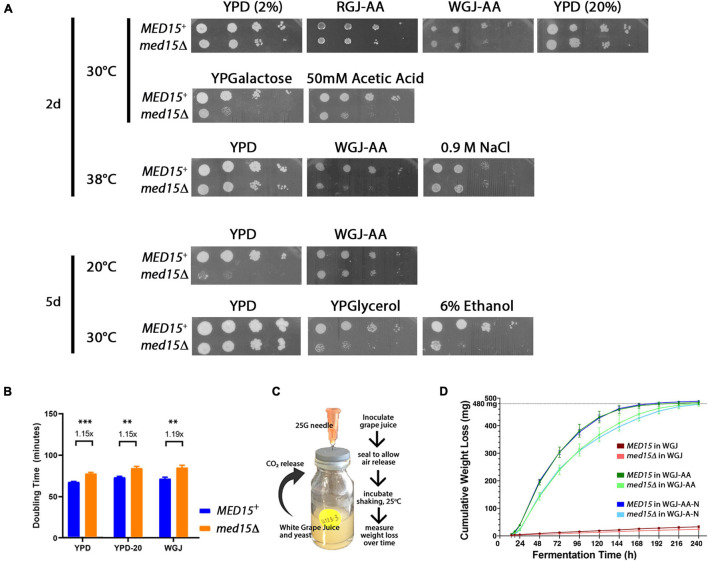
*Med15* growth and fermentation activities. **(A)** Phenotypic analysis using 10-fold serial dilutions of log-phase *MED15* and *med15Δ* cultures spotted on rich YPD media where D is glucose/dextrose added to 2% (2%) or 20% (20%) with or without supplements (ethanol, acetic acid), or on YP media with galactose (YPGalactose) or glycerol (YPGlycerol) or on amino-acid supplemented white or red grape juice media (20–21°Bx with added leucine, histidine, lysine, and uracil; (white grape juice, WGJ-AA; red grape juice, RGJ-AA) at the indicated temperatures (°C). **(B)** Growth, depicted as doubling times of WT (*MED15*) and *med15Δ* strains in rich media (YP) containing 2% glucose [YPD (2%)], 20% glucose [YPD (20%)], and supplemented white grape juice (WGJ) at 30°C. **(C)** Fermentation apparatus and experiment schematic. 1.25E7 cells were added to 5 mL of supplemented WGJ media in a small vial. A needle was inserted through the rubber seal to allow CO_2_ escape. Vials were incubated at 25°C in a shaking incubator and measurements of weight loss were made daily. **(D)** Cumulative weight loss over 7 days for WT and *med15Δ* strains in WGJ (no supplements), WGJ-AA (amino acid supplemented), and WGJ-AA-N (amino acid plus nitrogen supplemented). For panels **(B,D)**, data points are the averages of 3–6 biological replicates (transformants) and error bars are the standard deviation of the mean. Weight loss comparisons between genotypes were significantly different (*p* ≤ 0.01) at all time points in all media with the exception of 14 h in WGJ-AA (Supplementary Figure 1). Significance was determined using two-tailed *t*-tests.

The role of *MED15* in the growth of yeast in fermentation conditions was evaluated by examining the *med15*Δ mutant on plates prepared with white grape juice with amino acid supplementation (WGJ-AA) and other fermentation stressors. The effect of temperature was also evaluated since commercial fermentation reactions are typically carried out at 20–30°C for red wine and below 15°C for white wine (Reynolds et al., 2001). The *med15*Δ strain is cold-sensitive; it failed to grow at 14°C and grew poorly at 20°C (Figure 1A). Interestingly, the cold sensitivity of the mutant at 20°C was partially alleviated on WGJ-AA media. Conversely, the slight sensitivity of the *med15*Δ mutant to high temperatures was exacerbated in the presence of osmotic stressors such as grape juice (Figure 1A). The deletion strain also grew poorly relative to the wild type strain at 50 mM acetic acid (Figure 1A) and failed to grow on media containing 6% ethanol (Figure 1A). These concentrations were determined empirically and were chosen to give the greatest contrast in phenotype between the *MED15* and *med15Δ* strains. The acetic acid concentration of 50 mM is much higher than is normally present in wine while 6% ethanol is much lower than is normally present in wine.

Although grape juice media presents a relatively hostile environment even before cells begin the fermentation process, including nitrogen and amino acid limitation, high initial sugar concentration (hyperosmotic stress), and low pH between 2.9 and 3.9 (acid stress), the *med15* mutation did not specifically compromise growth in the grape juice environment (Figures 1A,B). Tolerance to high sugar media was confirmed by measuring growth rate in YPD with 20% instead of the usual 2% glucose (Figure 1B).

To determine the fermentation efficiency of the *med15Δ* mutant relative to the isogenic WT strain we conducted small-scale fermentation reactions (Figure 1C). Weight loss was measured over 8–10 days as a proxy for progression through the fermentation process, as weight loss is proportional to carbon dioxide produced by fermentation. In experiments conducted with agitation at 25°C, CO_2_ loss in the WT strain plateaued between 5 and 6 days (120–144 h) (Figure 1D). The *med15*Δ strain exhibited a marked delay at the start of fermentation with ∼40% less weight loss than WT lab strain during the first 24 h.

To more precisely define the specific fermentation deficiencies caused by the absence of the *MED15* gene, we determined the fermentation times at which the WT and mutant strains achieved comparable CO_2_ weight loss (Table 2 where T10 is time to 10% weight loss). The time to comparable weight loss were significantly different for all weight loss benchmarks evaluated (lag, t10, t35, t50, t80). For example, the fermentation lag time [time to 2 g/L weight loss (Peltier et al., 2018)] averaged 14.6 h in the WT strain compared to 18.8 h in the *med15*Δ strain (*p* = 4.24E-06).

**TABLE 2:** Equivalent Times to Weight Loss Benchmarks.

Time (h)	N^1^	Ave. cells per inoculum^2^	LAG^3^	T10 (h)^4^	T35 (h)	T50 (h)	T80 (h)
*MED15*	≥6	1.25E7	14.6	25.59	43.99	58.93	99.33
*med15* ^Δ^	6	1.25E7	18.8	29.63	54.84	73.66	133.8
P_*adj*_-value			4.2E-6	3.12E-3	1.86E-3	2.65E-3	3.12E-3

*^1^Biological replicates.*

*^2^5 mL WGJ-AA in a 10 mL vial, agitated at 175 rpm at 25°C.*

*^3^Lag is defined as the time to loss of 2 g/L CO_2_.*

*^4^Maximum CO_2_ weight loss = 487 mg; 10% was 48.7, 35% = 170.45, 50% = 243.5, 80% = 389.6 mg.*

We next investigated the effect of nitrogen availability on the slower fermentation kinetics in the mutant, by supplementing our standard fermentation media (WGJ-AA) with ammonium sulfate to increase the YAN from 242 to 434 mg N/L (WGJ-AA-N). There were no significant differences in weight loss for either the WT or mutant strain in either media type except at the earliest times (14 h, 18 h), where a small reduction in weight loss was observed for both genotypes in the nitrogen supplemented media (Figure 1D and Supplementary Figure 1). The time to equivalent weight loss benchmarks for the two strains was significantly different in WGJ-AA-N, just as it was in WGJ-AA media (Supplementary Figure 1).

In a companion experiment, we investigated the impact of providing extra sugar (22.1°Bx vs. 20.5°Bx) to the wild type and *med15*Δ mutant strain in the high YAN condition. The final (maximum) weight loss was increased in the wild type strain (528.4 mg versus 483.8 mg) 1.1-fold. In contrast, the mutant strain showed no comparable weight loss increase (0.98-fold) suggesting that *MED15* may play a modest role in sugar uptake or utilization (Supplementary Figure 1D). Interestingly, sugar consumption was incomplete regardless of genotype or media (Supplementary Figure 2).

### The Vulnerability of WT and *med15* Mutant Strains to Late Fermentation Stress

One explanation for the impact of the *med15Δ* on fermentation could be that there are fewer cells in the culture due to the increased doubling time, or that the strain undergoes progressive loss of viability under fermentation conditions. We therefore investigated to what extent the reduced fermentation rate in strains lacking *MED15* was a function of diminished culture saturation or sensitivity to accumulated stress in late fermentation (stationary phase). All fermentation reactions were inoculated comparably, with exactly 1.25 × 10^7^ cells from a fresh stationary phase culture being added to each vial.

**FIGURE 2:**
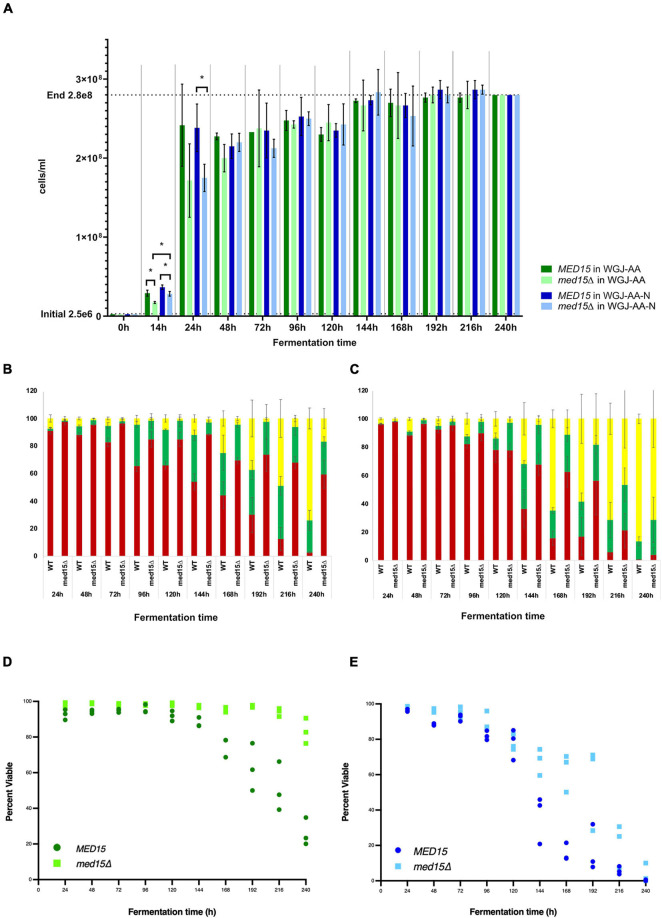
*MED15* mutants are less sensitive to late fermentation stresses. **(A)** Cell number was determined microscopically using a hemocytometer for the WT and *med15*Δ mutant in WGJ-AA and WGJ-AA-N media. **(B,C)** Results of FUN-1 staining with individual tallies of red (metabolically active), green (metabolically inactive), and yellow (permeabilized, dead) cells at each time point in WGJ-AA **(B)** and WGJ-AA-N **(C)** media. **(D,E)** Plots of viable cells (red + green) at times during fermentation in WGJ-AA **(D)** and WGJ-AA-N **(E)**. 3 biological replicates (transformants) are plotted for each time point for each genotype.

While the *med15Δ* strain does grow more slowly, cell density and cell count measurements reveal that the number of cells in the two strains is comparable by 48 h (Figure 2A). To determine if there are any differences in metabolic activity, cells growing in WGJ-AA or WGJ-AA-N were sampled every 24 h (sampling was from parallel punctured fermentation vials that were not being used for weight loss) with methylene blue or with the vitality dye, FUN-1 (Figures 2B–E). FUN-1 {[2-chloro-4-(2,3-dihydro-3-methyl-(benzo-1,3-thiazol-2-yl)-methylidene)-1-phenylquinolinium iodide]} which distinguishes between metabolically active, metabolically inactive and dead, permeabilized cells. To account for differences in nutrient availability and ethanol accumulation during fermentation at any particular time, comparisons of the two strains were made at times corresponding to CO_2_ weight loss benchmarks (Table 2) rather than at comparable times. The percentage of methylene blue staining cells (dead) trended higher in wild type cultures than in *med15*Δ cultures at benchmarks up to T80 (*P* < 0.1) but not later (Table 3). The conclusion using the FUN-1 stain supported these observations. Dead (permeabilized, yellow) and metabolically inactive cells started accumulating in mid-fermentation regardless of genotype (Figures 2B,C) with less than 25% of WT cells falling into the metabolically active category after day 4. Benchmark analysis showed that viable cells (red + green) were significantly elevated at T10, T50, and T80 benchmarks in the mutant relative to wild type (Table 3 and Figures 2D,E).

**TABLE 3:** Viability and Vitality in WGJ-AA at Weight Loss Benchmarks.

% Viability (MB)^1^	N^2^	T10^3^	T35	T50	T80	T98+
*MED15*	3	92.3	92.5	91.5	88.4	83.5
*med15*Δ	3	97.6	97.7	97.4	92.4	86.3
*P*-value		0.00960	0.0344	0.0419	0.0763	0.345

**% Vitality (FUN-1)^4^**	**N**	**T10**	**T35**	**T50**	**T80**	

*MED15*	3	90.9	88.64	85.72	65.61	
*med15*Δ	3	96.92	95.81	95.52	86.73	
*P*_*adj*_-value		3.0E-2	1.18E-2	1.18E-2	1.18E-2	

**% Viability (FUN-1)^5^**	**N**	**T10**	**T35**	**T50**	**T80**	

*MED15*	3	92.74	93.93	94.48	95.06	
*med15*Δ	3	98.51	98.64	98.13	97.63	
*P*_*adj*_-value		9.8E-2	2.9E-2	1.8E-2	0.196459	

*^1^(Total Cell Number - methylene blue stained cells)/Total ^∗^ 100.*

*^2^Biological replicates.*

*^3^Maximum CO_2_ weight loss = 487 mg; 10% was 48.7, 35% = 170.45, 50% = 243.5, 80% = 389.6 mg.*

*^4^[Total Cell Number - FUN-1 (yellow + green)]/Total ^∗^ 100.*

*^5^[Number of FUN-1 (red + green)]/Total ^∗^ 100.*

**FIGURE 3:**
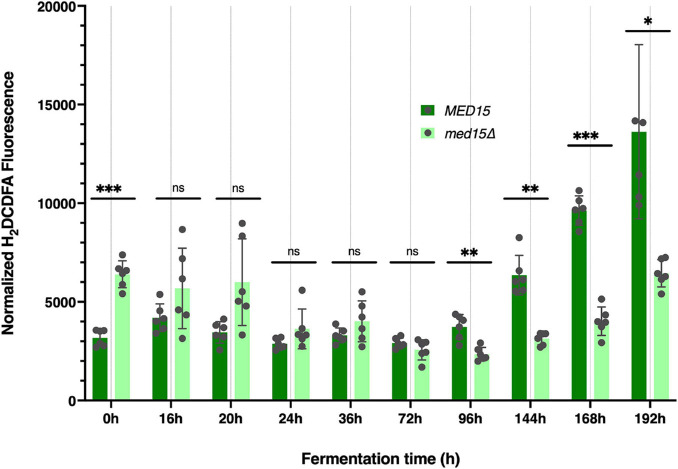
Reactive oxygen species are elevated at late times in fermentation specifically in the wild type strain. Reactive oxygen was measured at each time point in the fermentation time course and is shown as total arbitrary fluorescence units in H_2_DCDFA stained cells minus the fluorescence in unstained cells normalized to cell density. 3 biological replicates (transformants) and two technical replicates are shown; error bars are the standard deviation of the mean. Adjusted *P* values were determined with multiple two-tailed *t*-test and indicated as *, *p* ≤ 0.05; **, *p* ≤ 0.01; ***, *p* ≤ 0.001.

To determine if there was any impact of the *med15*Δ mutation on intracellular reactive oxygen species (ROS) which accumulate in response to rising ethanol levels and are partially attributable for the toxicity of the fermentation environment (Kitagaki et al., 2007) we measured ROS levels throughout the fermentation time course. Vials were opened at regular intervals for sampling. The first opening of the vial resulted in an ROS spike regardless of the specific time the vial was opened, and was likely artifactual. In the representative experiment shown in Figure 3, the spike occurred when the vial was opened at 8 h (not shown), but by 24 h the signal settled down, and was somewhat higher in the *med15*Δ strain than in WT. By 72 h the pattern began to change, with ROS produced by the WT strain outpacing that generated by the *med15*Δ mutant. When ROS was evaluated at equivalent weight loss benchmarks, only T80 and T98 were significantly different, with the level of ROS in the mutant being reduced relative to ROS in the wild types strain (Table 4).

**TABLE 4:** Reactive Oxygen Species and Ethanol Levels in WGJ-AA at Weight Loss Benchmarks.

ROS (AFU)^1^	N^2^	T10^3^	T35	T50	T80	T98+
*MED15*	6	2900	3300	3140	3220	9620
*med15Δ*	6	3680	3670	2950	2480	6460
*P*-value		9.63E-02	2.95E-01	3.14E-01	9.67E-03	1.71E-05

**EtOH (g/L)**	**N**		**T35^4^**	**T50**	**T80**	

*MED15*	6		33.32	44.98	68.39	
*med15Δ*	6		34.73	43.32	80.04	
*P*-value			4.0E-1	8.9E-1	2.0E-5	

*^1^Arbitrary Fluorescence Units normalized to OD_600_.*

*^2^Biological replicates.*

*^3^Maximum CO_2_ weight loss = 487 mg; 10% was 48.7, 35% = 170.45, 50% = 243.5, 80% = 389.6 mg.*

*^4^Maximum CO_2_ weight loss = 497 mg; 35% = 173.95, 50% = 248.5, 80% = 397.6 mg.*

Taken together, we found increased viability and decreased ROS accumulation in the *med15*Δ mutant. Hence, there is no evidence that the reduced fermentation rate in the *med15*Δ mutant is attributable to a lower number of viable cells and we speculate that the altered fermentation kinetics in the *med15*Δ mutant is due instead to an extensively altered transcriptome affecting many genes involved in the production and metabolism of ethanol.

### Ethanol Production Is Slower in the *med15* Mutant

**FIGURE 4:**
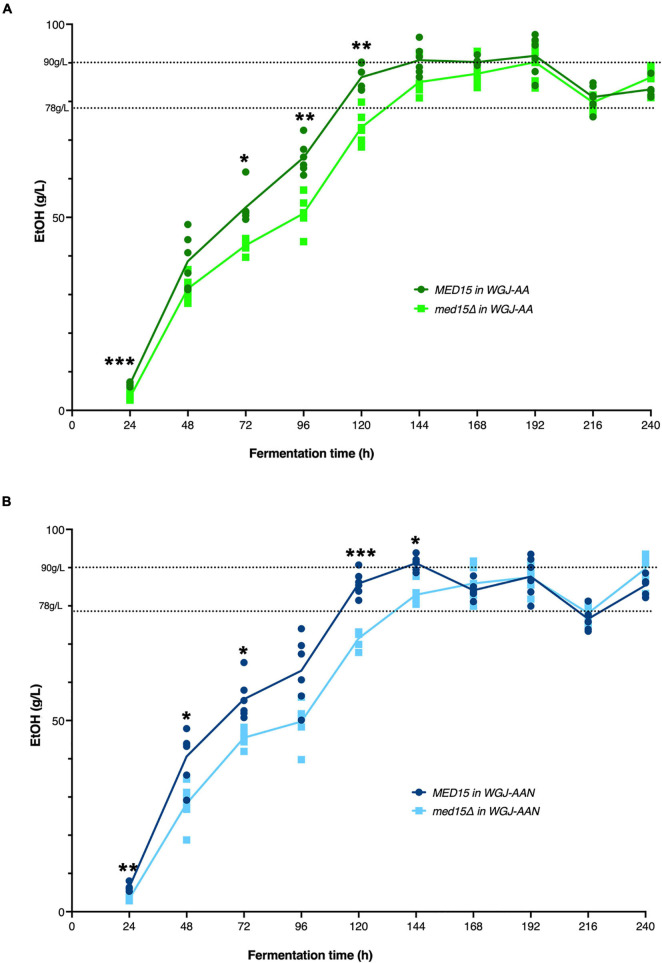
*MED15* mutants are defective in rate of ethanol production. **(A)** Direct measurements of ethanol production in the wild type and *med15*Δ strains over a 10-day fermentation time course in WGJ-AA or **(B)** WGJ-AA-N. Graphs show 3 biological and two technical replicates. Adjusted *P* values were determined with multiple two-tailed *t*-test and indicated as *, *p* ≤ 0.05; **, *p* ≤ 0.01; ***, *p* ≤ 0.001.

To investigate the impact of the *med15*Δ mutation on ethanol production, ethanol was measured over the fermentation time course. In the WT strain, ethanol levels peaked at approximately 6 days with approximately 90 g/L accumulated ethanol (Figure 4). As expected from the reduction in weight loss relative to the WT strain, the *med15*Δ strain produced less ethanol at the 6-day mark. When analyzed by CO_2_ weight loss benchmarks, the amount of ethanol produced at 35 and 50% weight loss is equivalent in the wild type and mutant strains, while the mutant has higher ethanol levels at the highest weight loss benchmark, perhaps due to fluctuation in the plateau phase of the curve (Figure 4) or previously noted defects in metabolism of non-fermentable carbon sources such as ethanol in the *med15*Δ mutant (Merz and Westermann, 2009; Bode et al., 2013; Table 4).

### Pre-treatment of the *med15*Δ Mutant With Low-Level Osmotic Stress Partially Suppresses the Fermentation Defect

**FIGURE 5:**
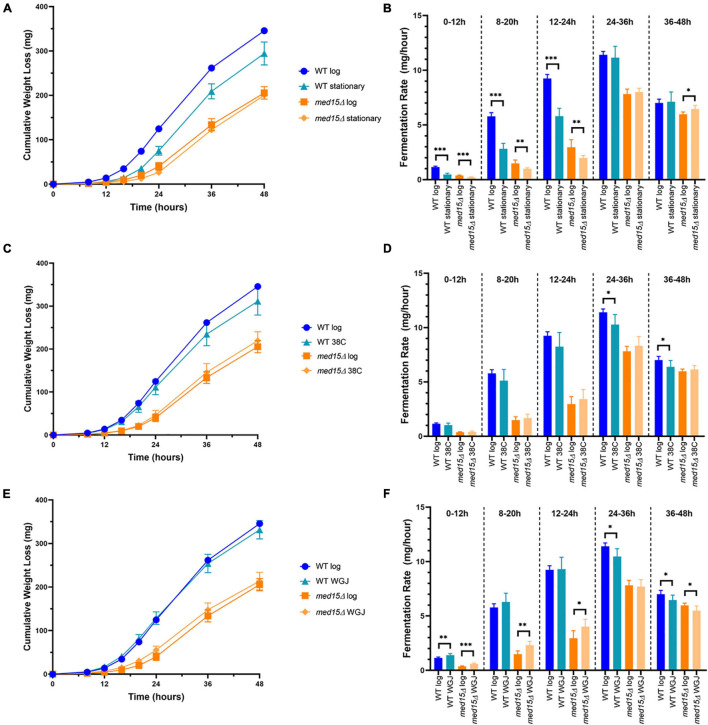
Fermentation in the *med15* mutant can be improved by related (same)-stress and cross-stress pre-treatments. **(A,B)** Log phase and stationary phase WT and *med15*Δ cultures were compared in a 6-day fermentation time course. Fermentation weight loss **(A)** and fermentation rate in 12-h time periods **(B)**. **(C,D)** The effect of cross-stress adaptation on fermentation rates was evaluated following a pre-treatment of log phase cells at 38°C. Fermentation weight loss **(C)** and fermentation rate in 12-h time periods **(D)**. **(E,F)** The effect of same-stress adaptation on fermentation rates was evaluated following pre-treatment of log phase cells with 1:3 WGJ:YPD media for 1 h. Fermentation weight loss **(E)** and fermentation rate in 12 h time periods **(F)**. Each data point is the average of 6 biological replicates and error bars reflect the standard deviation. The significance of differences between treated and untreated samples was determined using two-tailed *t*-tests. *, *p* ≤ 0.05; **, *p* ≤ 0.01; ***, *p* ≤ 0.001.

We next investigated whether changes in the *med15* fermentation profile might be due to a defect in adapting to the stresses inherent in the initial fermentation conditions. For example, the *med15*Δ mutant could be slower in activating stress response genes than WT, leading to a longer lag upon inoculation into grape juice media. To assess this, the length of time for the fermentation vial to begin losing weight was first compared using stationary phase versus log phase cells as inoculum. Stationary phase cells took longer than log phase cells to begin fermenting, and the lag was similar in both the WT and *med15*Δ strains, suggesting that the *MED15* gene is not required for exit from stationary phase (Figures 5A,B).

To further evaluate the role of *MED15* in adjusting to the initial exposure to grape juice media, we tested the response of the two strains to a same-stress or cross-stress pre-treatment. In addition to basal stress resistance that corresponds to the general Environmental Stress Response, resistance to future stress may be acquired following pre-exposure to a milder stress of the same or different type. The pre-exposure triggers relevant stress-activated response genes ahead of time (Berry and Gasch, 2008). Log phase cells were either exposed to a pre-treatment for 1 h or maintained in the initial growth conditions (SC-Leu media at 30°C) for an additional hour. Pre-treatment at 38°C (1 h) had no impact on the fermentation rate of the mutant strain (Figures 5C,D), but slightly impaired the response of the WT strain. This adverse effect is therefore *MED15*-dependent and may entail up or down-regulation of *MED15* target genes that compromise the response to fermentation conditions. In contrast to heat, pre-treatment with dilute grape juice to generate a mild osmotic stress increased fermentation in the *med15*Δ mutant at early timepoints (Figures 5E,F). Interestingly, the effect was minimal in the WT strain.

### Alcoholic Beverage Yeast *MED15* Alleles Can Improve Some Industrial Stress Responses

**FIGURE 6:**
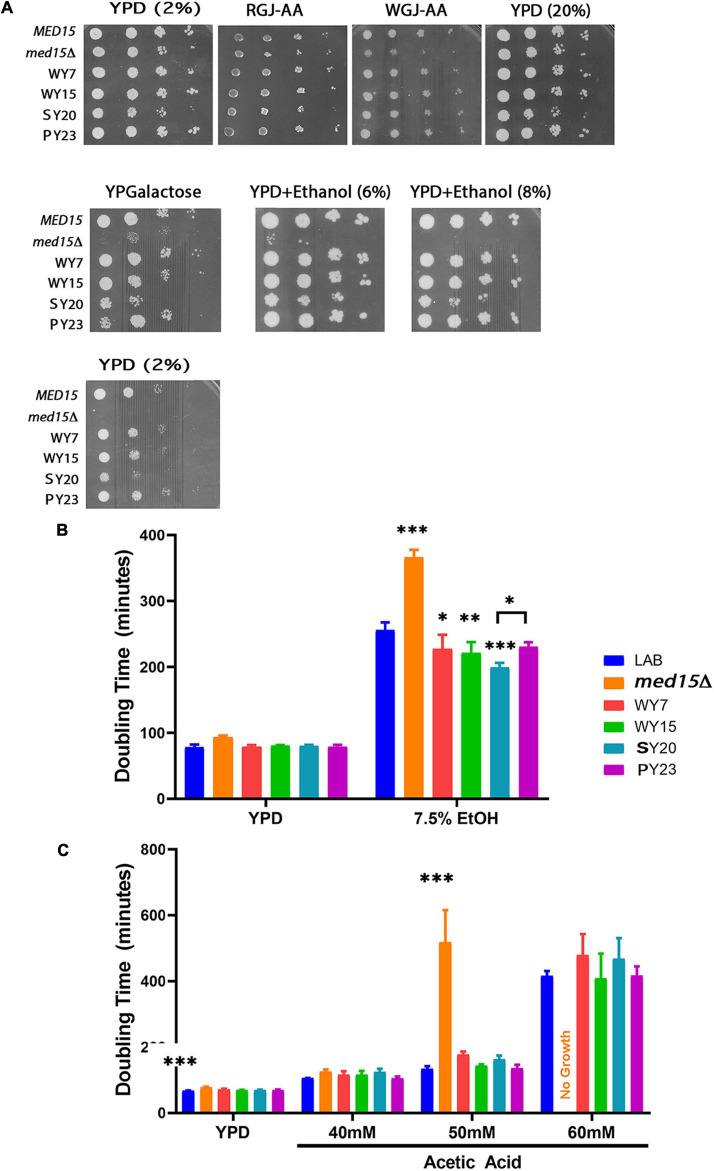
Alcoholic beverage *MED15* alleles complement all *med15* phenotypes and improve growth in acetic acid and ethanol. **(A)** Log-phase cultures of the *med15*Δ strain (OY320) carrying a CEN plasmid with the indicated *MED15* allele were serially diluted and spotted on YPD media with 2% or 20% glucose YPD media with 2% or 20% glucose (YPD (2%)) or (YPD (20%)), YPD (2%) with added ethanol (YPD+Ethanol (6%)) (YPD+Ethanol (8%)), YP media with 2% galactose instead of glucose (YPGalactose), supplemented red grape juice (RGJ-AA), and supplemented white grape juice (WGJ-AA) media. Incubation temperatures were as indicated. **(B,C)** Doubling times derived from growth curves of the *med15*Δ strain carrying different *MED15* alleles in YPD with or without indicated amounts of ethanol **(B)** or acetic acid **(C)**. For analyses in **(B,C)**, data points are the averages of 3 biological replicates (transformants) and error bars are the standard deviation of the mean. Significance was determined using ANOVA analysis with a Tukey *post-hoc* test. ^∗^, *p* ≤ 0.05; ^∗∗^, *p* ≤ 0.01; ^∗∗∗^, *p* ≤ 0.001.

Given the extensive impact of the Med15 Mediator subunit on the transcriptome (Ansari et al., 2012), together with its role in fermentation, we hypothesized that the *MED15* gene may have undergone specific sequence changes that contribute to the enhanced fermentation traits of wine yeast compared to LAB strains. To test this hypothesis, *MED15* was isolated from a set of strains associated with different alcoholic beverages (wine yeast WY7 and WY15, sake yeast SY20, and palm wine yeast PY23) (Table 5) and cloned into an expression plasmid between flanking upstream and downstream regulatory sequences from the S288C (LAB) *MED15* locus (Supplementary Figure 3). Since the diploid strains were homozygous at *MED15*, a single expression plasmid was constructed for each. Expression plasmids (Supplementary Table 1) were then introduced into a *med15*Δ derivative of the S288C LAB strain and tested for complementation of stress response phenotypes (Figure 6A). Each of the plasmids bearing *MED15* alleles from wine, sake, and palm wine yeast fully complement *med15*Δ phenotypes.

**TABLE 5:** Impact of *MED15* Alleles from Industrial Yeast on Times to Equivalent Weight Loss.

Time (h)	N^1^	Ave. no. cells per inoculum	T10^2^ (h)	T35 (h)	T50 (h)	T80 (h)
LAB	8	1.25E7	22.9	37.3	48.2	85.0
WY7	8	1.25E7	19.4^***^^3^	37.3	46.4	81.3
WY15	8	1.25E7	21.^0**^	36.7	44.7^***^	74.5^***^
SY20	8	1.25E7	21.1^*^	38.5	48.3	85.1
PY23	8	1.25E7	19.0^***^	36.2	44.5^***^	75.7^***^

*^1^Biological replicates.*

*^2^Maximum CO_2_ weight loss = 487 mg; 10% was 48.7, 35% = 170.45, 50% = 243.5, 80% = 389.6 mg.*

^3^Significance was assessed using two-tailed *t*-tests of comparisons of values for each *MED15*-WY allele vs. the LAB allele (*p < 0.05; **p < 0.01; ***p < 0.001).

To achieve more resolution and to examine the possibility that alcoholic beverage yeast *MED15* alleles confer an enhanced ability to cope with late fermentation stresses, the growth rate of the *med15* deleted LAB strain bearing *MED15*-LAB, *MED15-*WY, *MED15*-SY, or *MED15*-PY expression plasmids was evaluated in the presence of various concentrations of toxic byproducts of fermentation. Ethanol (Figure 6B) and acetic acid (Figure 6C) concentrations having a modest, rather than a devastating effect on growth of a strain with *MED15-*LAB and the expected deleterious impact on growth of a deletion strain were tested. Under these conditions, we found that strains carrying the SY20 allele were more robust than *MED15*-LAB in 7.5% ethanol, exhibiting a significant decrease in doubling time. No significant improvements in acetic acid tolerance were detected in comparisons of the industrial and LAB alleles at different concentrations of acetic acid (Figure 6C), however, the WY7 and SY20 alleles conferred more sensitivity than the LAB allele.

### Wine Yeast *MED15* Alleles Modulate the Fermentation Process

As seen in Figure 1, the *MED15* gene is required for efficient progression through the fermentation process. Before specifically investigating the role of *MED15* in WY fermentation, we first compared the fermentation profile of the diploid yeast strains to the haploid LAB strain with the native LAB *MED15* allele. Three of the diploid strains outpaced the LAB strain at all time points and one diploid strain (SY20) outpaced the LAB strain primarily in the first 48 h. Interestingly, the final weight loss in the diploid strains was equivalent to that of the haploid LAB strain (Supplementary Figure 4) suggesting that while the industrial strains are better fermenters than the LAB strain, it is a matter of rate, and not the extent to which the sugar in the grape juice can be metabolized.

**FIGURE 7:**
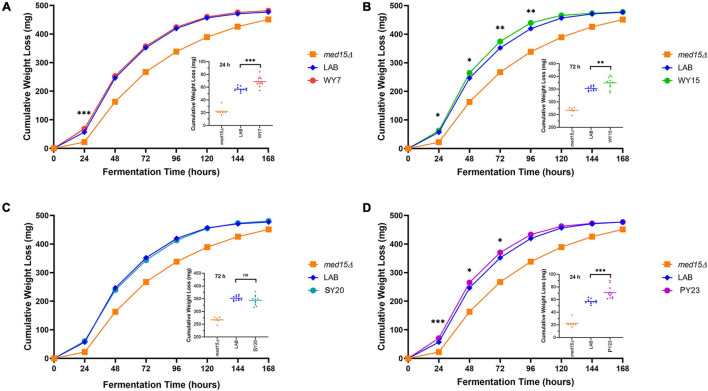
Fermentation of white grape juice is boosted by *MED15* alleles from alcoholic beverage yeast. **(A–D)** Relative cumulative weight loss in WGJ fermentation experiments for the *med15Δ* strain (OY320) carrying plasmids expressing the LAB (S288C) or *MED15*-WY/SY/PY alleles. Fermentations were conducted at 25°C with constant agitation as shown in Figure 1C. For all panels, data points are the averages of 10 biological replicates (transformants) and error bars are the standard deviation of the mean. Significance was determined using two-tailed *t*-tests. ^∗^, *p* ≤ 0.05; ^∗∗^, *p* ≤ 0.01; ^∗∗∗^, *p* ≤ 0.001. Insets depict the distribution of data points for the most significant time point.

We next investigated to what extent the *MED15*-WY, SY and PY alleles contributed to the improved fermentation profile. Since the *MED15* gene is potentially only one of a large number of genes with adaptive changes in the industrial yeast, we did not anticipate large changes. However, the impact was significant for three out of four of the *MED15* alleles. Only one allele (SY20) failed to improve on the *MED15*-LAB fermentation profile (Figure 7). The specific impact of the *MED15* allele varied. The WY7 allele increased weight loss relative to LAB during the first 24 h of fermentation (day 1) while the WY15 and PY23 alleles increased weight loss mid-fermentation (days 2–5) (Figure 7). Comparisons using weight loss benchmarks confirm these observations (Table 5).

### Laboratory and Alcoholic Beverage *MED15* Alleles Differ in Polyglutamine Tract Lengths and Non-synonymous SNPs

One feature of the Med15 amino acid sequence is the overrepresentation of the amino acid glutamine (Q). The bias is especially striking at three positions in the protein where uninterrupted glutamine and glutamine-alanine tracts are found (Cooper and Fassler, 2019). The length of the Q tracts within *MED15* alleles is known to vary amongst different *S. cerevisiae* strains (Cooper and Fassler, 2019) with potential enrichment in certain tract lengths among the European wine yeast (Cooper and Fassler, 2019). The *MED15* alleles from the four industrial strains initially examined in this study were re-sequenced using Sanger technology to ensure high accuracy through repetitive sequences (Table 6). WY7 has tract lengths typical of European WY strains with the N terminal most tract (Q1) having a length of 21 glutamines, the central tract (Q2, alternating QA tracts with an interruption of 1 or 2 QAA motifs) having a length of 29, and the C terminal most tract (Q3) having a length of 25. WY15 shares Q2 and Q3 tract lengths with other European wine strains, but has a longer Q1 tract of 28. SY20 and PY23 in our study are strains used in the production of sake and palm wine, respectively, and are not members of the European wine yeast clade. They each have a Q1 tract length of 21 like WY7 but a Q2 of 28 and Q3 of 27 in the sake yeast (SY20), and a Q2 of 12 and a Q3 of 27 in the palm wine yeast (PY23) (Table 6).

**TABLE 6:** Yeast *MED15* Polymorphisms.

Strain	Designation	Niche/use(s)	Single nucleotide polymorphisms		Q1^3^	Q2^4^	Q3^5^
LAB	S288C	Research	Synonymous^1^	Non-Synonymous^2^	12	10(2)17	23
WY1A	Lalvin 71B-1122	Red and white	A2532C, T2922C	K98N, T322I, V944L	12	10(2)16	13
WY1B			T2109G, T3210C	K98N, A713T	20	9(2)17	25
WY1C			C204T, T2109G, A2532C, T2922C, T3210C	L28H, T322I, A713T, V944L	12	10(2)16	25
WY2	Lalvin RC-212 Bourgovin	Red	T2109G, T3210C	K98N, A713T	20	11(1)17	25
WY3	Lalvin ICV D-47	White	T903C, T2109G, T3210C	K98N, A713T, R868C	21	9(2)17	25
WY4	Lalvin EC 1118	Champagne	T2109G, T3210C	K98N, A713T	21	9(2)17	25
WY5	Lalvin K1-V1116	White	T2109G, T3210C	K98N, A713T	21	9(2)17	25
WY7	Lalvin ICV D254	Red and white	T2109G, T3210C	K98N, A713T	21	11(1)17	25
WY15	DSM Fermichamp	Fermentation booster	T2109G	A49V, K98N, M658L, A713T, S774N	28	10(1)18	25
SY20	Kyokai 7	Sake	C204T, G678A, T1011C, C1020T, G1965A, C2160T, A2532C	K98N, P279L, V513I, A561T, N727Y, V944L	21	10(2)16	27
PY23	NRRL Y-17772	Palm wine	C204T, G300A, G486A, T1011C, C1020T, A2532C	K98N, S111Y, V944L	21	12(0)0	27

*^1^Changes to the nucleotide sequence relative to the S288C LAB sequence at the indicated coordinate with no impact on the amino acid sequence.*

*^2^Changes to the nucleotide sequence relative to the S288C LAB sequence resulting in an amino acid substitution at the indicated position.*

*^3^The glutamine tract length at the Q1 position.*

*^4^The sequence at the Q2 position represented in three parts as the number of glutamine-alanine (QA) repeats followed by the number of glutamine-alanine-alanine (QAA repeats) shown in parentheses and finally the number of glutamine-alanine repeats on the other side of the alanine tract.*

*^5^The glutamine tract length at the Q3 position.*

To increase our sampling of the European wine yeast clade, we determined the DNA sequence of *MED15* from five additional European wine yeast strains (Table 6). WY1 was found to be triploid for *MED15* while WY2, WY3, WY4, and WY5 are homozygous diploids. WY2-5 have a Q1 of 20–21, while WY1 has two Q1 alleles of 12 like the S288C strain, and another of 20. All five additional WY have at least one allele with a Q3 of 25. These patterns are consistent with our earlier observation (Cooper and Fassler, 2019) that European WY *MED15* alleles are enriched in a Q3 track length of 25. In addition to the variation in glutamine tract genotypes, the WY *MED15* alleles have 2–6 non-synonymous SNPs (nsSNPs) affecting non-glutamine parts of the protein as well as 1–7 synonymous SNPS (Table 6 and Supplementary Figure 5).

In earlier computational work (Cooper and Fassler, 2019) we found that the preponderance of European wine yeast have a Q3 tract length of 25. Both WY7 and WY15 have Q3 tracts of 25 and both boost fermentation relative to the LAB allele although with different patterns (Figure 7 and Table 5). To further test the relevance of Q3 tract length, we examined the impact of 5 additional European wine yeast alleles with a natural Q3 tract length of 25 on fermentation performance of the LAB *med15*Δ strain. Except for the allele from WY2, these strains exhibited no improvement over the LAB *MED15* allele in fermentation experiments, suggesting that Q3 tract length is unlikely to be the sole determinant of fermentation activity (Supplementary Figure 6).

### Fermentation Characteristics of Clean *MED15* Alleles Differing From Laboratory Alleles in Q Tract Lengths

**FIGURE 8:**
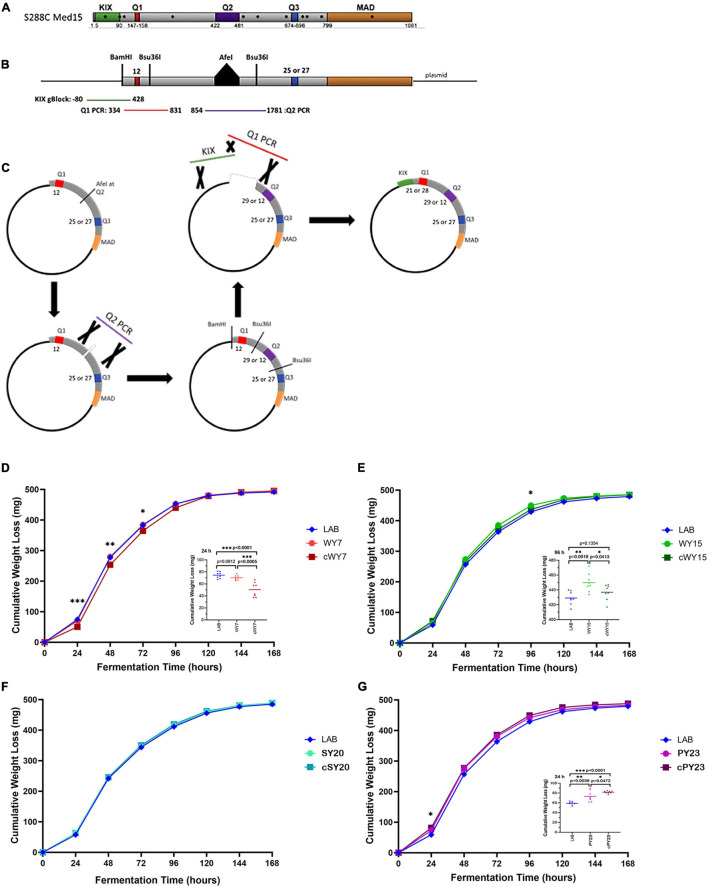
Both glutamine tracts and non-glutamine SNPs are involved in the fermentation booster phenotypes seen in strains carrying WY *MED15*. **(A)** The S288C (LAB) Med15 protein is shown in cartoon form with the positions of the three glutamine tracts between the N terminal KIX domain and the C terminal MAD domain indicated. Also shown with asterisks are the positions of non-synonymous SNPs in the wine yeast MED15 alleles evaluated in this study. **(B)** A second cartoon of a synthetically reconstructed S288C Med15 protein that lacks the KIX domain and has an *Afe*I site at the normal position of the Q2 tract and have either 25 or 27 glutamines at Q3. PCR fragments are shown to indicate the region amplified from the *MED15*-WY alleles that were designed to encompass the Q tract without nearby SNPs. **(C)** The process by which “clean” WY alleles were constructed from the parts illustrated in panel **(B)**. **(D–G)** The fermentation profiles of the LAB allele, the original alcoholic beverage alleles (*e.g*., WY) and the “clean” (*e.g*., cWY) alleles are plotted. Data points are the averages of 8 biological replicates (transformants) and error bars are the standard deviation of the mean. Significance was assessed using a two-tailed *t*-test. Insets depict the distribution of data points for the most significant time point.

To examine the relevance of the Q tract differences versus nsSNPs in LAB and industrial *MED15* alleles, synthetic “clean” (c) *MED15* genes were constructed (Figures 8A–C) lacking most or all non-synonymous changes. Strains with the clean alleles were characterized in fermentation assays side by side with examples of the original allele as well as the LAB allele as seen in Figures 8D–G and as summarized in cartoon form in Table 7.

**TABLE 7:** Cartoon Summary of *MED15* alleles from Alcoholic Beverage Yeasts with and without Non-synonymous SNPs^1^.

Strain	SNPs	Q-tract polymorphisms			Fermentation Phenotypes	
		Q1	Q2	Q3	WY relative to LAB	Clean relative to original
LAB	Non-synonymous	12	10(2)17	23		
WY7	K98N, A713T	21	11(1)17	25	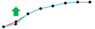	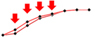
cWY7	A713T	21	11(1)17	25		
WY15	A49V, K98N, M658L, A561T, S774N	28	10(1)18	25	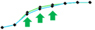	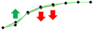
cWY15	None	28	10(1)18	25		
SY20	K98N, P279L, V513I, A561T, N727Y, V944L	21	10(2)16	27	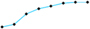	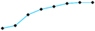
cSY20	None	21	10(2)16	27		
PY23	K98N, S111Y, V944L	21	12(0)0	27	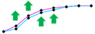	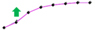
cPY23	None	21	12(0)0	27		

*^1^This table summarizes in cartoon form the relative fermentation curves for strains with the LAB *MED15* allele compared to *MED15* alleles from alcoholic beverage yeasts and for strains with the industrial allele compared to the same allele minus many or all nsSNPS.*

*The data summarized here is from Figures 7, 8.*

Strains with the cWY7 [Q1 = 21, Q2 = 11(1)17, Q3 = 27] allele from which only one (K98N) of two non-synonymous SNPs was removed performed more poorly than the original allele (Figure 8D and Table 7) over several time points. The possibility that K98N might be a key SNP for fermentation performance is consistent with the pattern in the cWY15 allele which lacks all 5 SNPs including K98N and in which mid-profile performance is also diminished. However, the importance of the K98N allele is contradicted by the cPY23 allele, which lacks 3 of 3 SNPs including K98N and in which we see no reduction in fermentation performance. Furthermore, the fermentation performance of both the cWY15 and the cPY23 alleles is improved early in fermentation (48 h) in the absence of nsSNPs suggesting that certain mutations may prevent the full impact of the Q tracts early in the fermentation time course. The remainder of the cPY23 fermentation time course is not affected by the absence of nsSNPS, clearly indicating that the Q tracts are important to the PY23 fermentation booster phenotype.

## Discussion

As one of the subunits of RNA Polymerase II Mediator tail module, *MED15* is well known for its role in regulating expression of stress response and metabolic genes. However, no previous research has directly investigated the role of *MED15* in the fermentation process. Here we show that the *MED15* subunit of the Mediator complex is important for the efficient fermentation of grape juice. Despite the mutant’s impaired tolerance to acute exposure to various chemicals (notably ethanol and acetic acid), the *med15*Δ strain is not sensitized to late-stage fermentation stresses presumably because they have accumulated slowly. Instead, the fermentation phenotype likely reflects reduced gene expression of key metabolic genes including those involved in ethanol production and metabolism. In addition, we have shown that some *MED15* alleles differing in poly-Q tract lengths and SNPs confer more efficient grape juice fermentation, which is consistent with the hypothesis that *MED15* alleles from wine yeast may be optimized for fermentation. Future experiments conducted in industrial strains and directly assessing fitness will be needed to confirm the importance of *MED15* in commercial wine making environments and the adaptive potential of alleles found in alcoholic beverage yeasts. Nonetheless, the improvements afforded by the WY and PY *MED15* alleles that we have documented in this study are novel and surprising and provide important groundwork for pursuing more targeted experiments with industrial implications.

### *med15*Δ Does Not Sensitize Yeast Strains to the Gradual (Cumulative) Stresses of Late Fermentation

Although we and others have shown that *med15*Δ strains are highly sensitive to acute ethanol and acetic acid stress (Figure 1; Kawahata et al., 2006; Qian et al., 2012), there appears to be no excess sensitivity to these compounds in the late fermentation environment, presumably because the adaptation of yeast to the gradual accumulation of acetic acid and ethanol during fermentation involves different pathways.

Yeast cells are known to lose metabolic activity and cell membrane integrity in the presence of acetic acid (Rego et al., 2014). Unexpectedly, we found that the loss of cell viability and increased permeability in *MED15*+ strains during late-stage fermentation appears to be curtailed in the *med15*Δ deletion strain. The altered transcriptome in the *med15* mutant may influence the sensitivity of the mutant cells to acetic acid or it may influence the accumulation of acetic acid. It is possible that the reduced expression of *MED15* target genes that account for deficiencies in respiratory growth in the mutant may reduce acetic acid levels and hence ROS and cell death.

Since both acetic acid and ethanol are known inducers of apoptosis (Ludovico et al., 2001; Kitagaki et al., 2007), *MED15* might have a pro-apoptotic effect by virtue of regulating the expression of the appropriate genes. The intersection of genes regulated by *MED15* (Hu et al., 2007) and those having apoptosis annotation includes a short list of candidates (Supplementary Figure 7). Mutations in eight genes that are positively regulated by Med15 are annotated as reducing apoptosis phenotypes. These may be important in the pro-survival phenotype in the *med15*Δ mutant.

### Survival of the *med15* Mutant in Late-Stage Fermentation

The *med15*Δ mutant fails to grow on non-fermentable carbon sources and is thus compromised in oxidative metabolism (Merz and Westermann, 2009; Bode et al., 2013). Hence, the CO_2_ weight loss and ethanol production profiles of the *MED15*+ and *med15*Δ mutant strains are somewhat affected by different constraints. In the *MED15*+ strain, weight loss is the sum of the anaerobic fermentation process plus any residual ongoing respiration, both leading to the production of CO_2_, followed by additional CO_2_ production after the diauxic shift (upon accumulation of ethanol) whereupon cells begin to catabolize ethanol and the other carbon compounds *via* the TCA cycle and oxidative phosphorylation in the mitochondria. The diauxic shift is accompanied by elevated reactive oxygen species (ROS) levels and an accompanying oxidative stress response (Bisson and Walker, 2015). In contrast, the *med15* mutant presumably produces CO_2_ primarily by fermentation, and less so by respiration, since it is not fully metabolizing available ethanol. Using H_2_-DCFDA, a widely used fluorescent dye for measuring intracellular ROS levels, we found that the mutant produces less ROS during late fermentation and this may account for the reduced mortality. The apparent discrepancy between the observation that WT and *med15*Δ strains consume ethanol equivalently late in fermentation (Figure 4) and the observation that levels of ROS are lower in the *med15*Δ mutant at that time (Figure 3) are potentially explained by normal conversion of ethanol to acetaldehyde in the mutant, followed by reduced activity in the later steps leading to acetyl CoA and subsequent respiration-mediated CO_2_ production. The oxidation of acetaldehyde to acetate is catalyzed by both cytosolic and mitochondrial aldehyde dehydrogenase, of which the main isoforms (Ald6 and Ald4, respectively) are important for growth on ethanol. Our preliminary q-RT-PCR and RNA-Seq experiments show that the *ALD4*, *ALD5*, and *ALD6* genes are positively regulated by *MED15* and may be rate limiting in the mutant.

### Fermentation Stress Response

We examined the sensitivity of the *med15Δ* mutant to cross-stress pre-treatments to determine whether the slower fermentation response in the mutant might relate to deficiencies in the ability of the mutant strain to rapidly mount a stress response. Pre-exposure to heat stress had no impact on the fermentation rate of the mutant strain (Figures 5C,D), but impaired the response of the WT strain. This adverse effect is therefore *MED15*-dependent and may entail up or down-regulation of *MED15* target genes that compromise the response to fermentation conditions. When strains were pre-exposed to dilute grape juice generating a mild osmotic stress, fermentation was increased in the *med15*Δ mutant at early timepoints (Figures 5E,F) but was minimal in the WT strain suggesting that *MED15* may be repressing genes that can, but do not normally contribute to adaptation. The results of the pre-treatment experiments suggest that the fermentation defect in the mutant is unlikely to be due to its known deficiencies in stress response and is more likely the consequence of changes in the expression of metabolic genes involved in the production or consumption of ethanol.

In addition to the general and stimulus-specific response mechanisms which are mounted transiently in cells experiencing stress (Gasch et al., 2000; Causton et al., 2001), sustained changes in transcript abundance over fermentation time courses of up to 15 days are also observed (Marks et al., 2008), indicative of an adaptive response to fermentation stress. Of the 223 fermentation stress response (FSR) genes, exhibiting sustained and dramatic induction throughout the entire time course, ∼25% overlap with *MED15* targets identified under laboratory conditions (Hu et al., 2007; Supplementary Figure 7) including 43 that are positively and 8 that are negatively regulated. Consistent with this, our preliminary RNA-Seq data suggests that in addition to Med15 regulation of glycolytic and alcoholic fermentation genes during growth in laboratory culture conditions (YPD), there is a set of metabolic genes that are modulated by Med15 specifically during grape juice fermentation. The presence of hexose transporter genes in the fermentation-specific differentially regulated gene set together with our observations of differential sugar utilization in the *med15*Δ mutant (Supplementary Figure 1D) suggests that the defects in fermentation efficiency in the mutant may be due in part to a reduced rate of sugar uptake. Many Med15 regulated fermentation specific genes also influence the efficiency of amino acid anabolism. In the nitrogen-limiting grape juice environment, the ability to modulate the production of amino acids may allow for more efficient fermentation.

### Fermentation and Glutamine Bias in Med15

We find that *MED15* alleles originating in strains used in production of alcoholic beverages are somewhat more efficient in sugar utilization and ethanol production. With the polyglutamine tracts within Med15 being the primary difference among alleles, we hypothesized that specific tract lengths and/or tract length combinations could be important determinants of a fermentation boosting/permissive transcriptome. We analyzed seven alleles (WY1-5, WY7, and WY15) belonging to the European wine clade, as well as one sake yeast (SY20) and one palm wine yeast (PY23) which are phylogenetically distinct (Strope et al., 2015).

Based on the analysis of the *MED15* sequence reported for 150 strains (Cooper and Fassler, 2019) and on the resequencing of the *MED15* allele from the nine strains described (Supplementary File 1), we hypothesized that a Q3 tract of 25 glutamines (compared to a Q3 tract of 23 glutamines in the LAB allele) is an important sequence feature in wine yeast strains that might contribute to the increased fermentation rate we observed when certain of the WY *MED15* alleles are examined in the LAB strain. Of the 7 European alleles, only WY15, WY7, and WY2 have any impact on fermentation, while WY1 and 3–5 alleles behave similarly to the LAB allele during WGJ fermentation. At face value, these observations suggest that a Q3 tract of 25 glutamine residues, is insufficient on its own to change the course of a fermentation reaction and that the WY2, WY15, and WY7 fermentation phenotype may instead be a function of Q3 length in combination with other Q tract lengths or with non-synonymous SNPs in the sequence.

Synthetic *MED15* genes were used to evaluate the role of Q tract length combinations in the absence of all other amino acid substitutions that could be expected to influence the fermentation phenotype (Figures 8D–G, Table 7, and Supplementary Figure 5). Strikingly, the removal of SNPs from the PY23 *MED15* allele had little effect on the fermentation profile, confirming the importance of the Q tracts. The PY23 *MED15* sequence is distinctive in two ways. It has a Q3 tract length of 27, 2 residues longer than any of the European wine yeast. In addition, it has an unusual Q2 tract. At 12 QA repeats, it is only half the length of the LAB or any other variant allele. Whether this short Q tract is primarily responsible for the changes in fermentation profile, or whether it works in collaboration with the Q1 of 21 and/or the Q3 of 27 will require additional investigation. These observations suggest that individual tract lengths or combinations of specific tract lengths may correspond to specific industrial phenotypes.

### What Is the Molecular Basis of Glutamine Tract-Dependent Function in Med15?

The molecular mechanism for the glutamine tract length dependence of fermentation could be a direct effect due to glutamine dependent interactions between Med15 and any of its transcription factor or chromatin associated interactors. Alternatively, the effect could be indirect. We speculate that the Q tracts may not themselves mediate these protein-protein interactions, but instead interact with one another to generate specific Med15 conformations that contribute to a more or less active Mediator complex. Further tests of this hypothesis and additional characterization of the Med15 protein will be necessary to understand the details of this mechanism.

## Data Availability Statement

The raw data supporting the conclusions of this article will be made available by the authors, without undue reservation.

## Author Contributions

DC and YJ conducted experiments, analyzed data, and participated in editing drafts of the manuscript. SS and LW conducted experiments. JF designed the experiments and participated in supervision and writing. All authors approved the final version of the manuscript.

## Conflict of Interest

The authors declare that the research was conducted in the absence of any commercial or financial relationships that could be construed as a potential conflict of interest.

## Publisher’s Note

All claims expressed in this article are solely those of the authors and do not necessarily represent those of their affiliated organizations, or those of the publisher, the editors and the reviewers. Any product that may be evaluated in this article, or claim that may be made by its manufacturer, is not guaranteed or endorsed by the publisher.

## Footnotes

1
https://nrrl.ncaur.usda.gov/
