# Supplementary material for: Possible Role for Allelic Variation in Yeast MED15 in Ecological Adaptation
Source: Front Microbiol. 2021 Oct 18;12:741572. doi: 10.3389/fmicb.2021.741572 (PMC8558680; doi:10.3389/fmicb.2021.741572)
Supplement: Supplementary file 1 [file Data_Sheet_1.pdf]

Supplementary Material

File	Description	Page
**File S1**	Description of the wine yeast strains used in this study	**2**
**File S2**	Plasmid construction details	**4**
**Figure S1**	Effect of YAN on fermentation	**6**
**Figure S2**	Sugar consumption is comparable but incomplete in WGJ-AA and WGJ-AA-N	**8**
**Figure S3**	Construction of *MED15* expression plasmids	**9**
**Figure S4**	Comparison of the haploid *MED15* strains compared to the original diploid alcoholic beverage strains in a fermentation assay	**10**
**Figure S5**	*MED15* alleles from alcoholic beverage yeast differ from the *MED15*-LAB by the length of the Q tracts and by a small number of non-synonymous SNPs	**11**
**Figure S6**	Additional European WY share fermentation kinetics with WY7	**13**
**Figure S7**	Subsets of genes regulated by *MED15* that may play a role in late fermentation	**14**
**Table S1**	Plasmids used in this study	**15**
**Table S2**	Primers used in this study	**18**
	Literature Cited	**19**

File S1. Description of yeast strains used in this study

WY1-5 and 7 are commercial strains from Lallemande Inc. WY1 (Lalvin 71B, Narbonne, France) is known for the production of the ester isoamyl acetate (3-methylbutyl acetate) which has a banana/pear aroma and produces high levels of glycerol, contributing to the mouthfeel effect of this yeast. WY2 (Bourgorouge RC212™, Burgundy region of France) was selected for its ability to ferment a traditional heavier style Burgundian Pinot Noir and serves as the 'reference yeast' for the making of quality Pinot Noir due to its elevated anthocyanin content, and limited adsorption of polyphenols on yeast cell walls, thus limiting color loss and maintaining structure during aging. WY3 (Lalvin ICV D-47, Côtes du Rhône region in France) confers enhanced aroma and flavor, partially attributable to high β-glucosidase activity and the accumulation of polysaccharides in the must during fermentation which stabilize aromatic compounds. WY4 (Lalvin EC-1118, Champagne region of France) has a neutral sensory contribution and is known for its excellent properties in producing base wine for Champagne as well as "in-bottle" secondary fermentation. WY5 (Lalvin K1-V1116, France) produces esters (isoamyl acetate, hexyl acetate, phenyl ethyl acetate) conferring fresh floral aromas to neutral varieties or high yield grapes and is resistant to difficult fermentation conditions such as low turbidity, low temperature, and low fatty acid content. WY7 (Lalvin ICV D-254, Rhône Valley, France) was isolated from a Rhône Valley Syrah fermentation and has an alcohol tolerance of up to 16% (v/v) when the fermentation is aerated and the temperature is maintained below 28°C (https://scottlab.com/icv-d254-yeast-d254). Finally, WY15 (DSM Fermichamp, Narbonne, France) is used primarily as a booster in stuck fermentations. Since *S. cerevisiae* is a glucophilic yeast, preferring glucose over the fructose in grape must, the glucose is consumed first, causing the proportion of fructose to increase as fermentation progresses. The low fructose utilization capacity of *S. cerevisiae* may lead to sluggish or stuck fermentations. The Fermichamp strain has an altered low affinity hexose transporter gene, *HXT3*, resulting in higher fructose utilization than standard *S. cerevisiae* wine strains, and thus accounting for its booster phenotype (Guillaume *et al.*, 2007; Zuchowska *et al.*, 2015). SY20, Kyokai 7 or K7 group strains, isolated from a sake brewery in Nagano Prefecture, Japan, in 1946, are used in production of sake, an alcoholic beverage made from steamed rice. The final alcohol concentration of sake mash reaches nearly 20% and the ability of the K7 sake yeast to tolerate high ethanol (Fig. 6B) is caused by loss-of-function mutations in *RIM15*, *MSN4*, and *PPT1* genes (Watanabe *et al.*, 2011; Noguchi *et al.*, 2012; Watanabe *et al.*, 2012) which

encode proteins involved in transcriptional regulation of stress response genes via the stress response (STRE) and heat shock elements (HSE). Thus, K7 group strains show lower stress tolerance than laboratory strains (Urbanczyk *et al.*, 2011). Finally, the PY23 strain (NRRL Y-17772, ARS collection) is a palm wine yeast. Palm wine is a popular alcoholic drink in Nigeria and West and Central African obtained by fermenting the sugary sap of the oil palm tree, *Elaeis guianeensis* or of raffia palms (*Raphia hookeri* and *Raphia vinifera*) (Ezeronye and Legras, 2009).

File S2. Plasmid construction details

***MED15* plasmids.** Plasmids used in this study are summarized in **Table S1** along with their source. *MED15* alleles were isolated from diploid yeast strains and expressed on plasmids containing lab strain *MED15* regulatory sequences (pYJ2155-58, and pYJ2166-73). The lab strain (BY4742) allele (LAB) was expressed from the same plasmid backbone (pDC2141). Wine yeast alleles were PCR amplified from diluted genomic DNA using Q5 polymerase, 2x Master Mix and the primers *MED15* F-245 and *MED15* R+3498 at a final concentration of 0.5µM. The thermocycler program was: 1x (98°C for 30 seconds), 30x (98°C for 10 seconds, 63°C for 30 seconds and 2°C for 2 minutes 20 seconds), and 1x (72°C for 2 minutes). Selected primers were complementary to sequences outside of the *MED15* coding sequences, allowing for the upstream and downstream homology to be used in the gap repair of the mini-*GAL11*(*MED15*) plasmid (pRS315 M-WT) (Kim *et al.*, 2008) from which the *MED15* coding sequence had been removed by digestion with *Bam*HI and *Spe*I. All gap repair reactions were carried out in the *med15Δ* deletion strain. Successful gap repair was determined by the simultaneous PCR amplification of two regions (primers: *MED15* F+384, *MED15* R+529, *MED15* F+1924, and *MED15* R+2143) including sequences encoding the variable length polyglutamine tracts. The shorter tract lengths in the LAB allele were easily distinguished from other *MED15* alleles after electrophoresis on 4% (1x TAE) agarose gels. Where the wine yeast strain was heterozygous at *MED15*, both alleles were isolated. Plasmids were isolated from yeast strains using a modification of the Zymoprep Yeast Plasmid Miniprep II kit (Zymo Research, D2004). Rescued plasmids were transformed into competent DH5α cells (Chan *et al.*, 2013). Individual bacterial transformants were isolated (pYJ2155-58, and pYJ2166-73). Plasmids were isolated from these bacteria using a commercial miniprep kit (Qiagen) or by alkaline lysis and ethanol precipitation (Birnboim and Doly, 1979). The sequence of each isolated allele and correct construct structure was determined by Sanger sequencing.

Synthetic (SNP-free, clean) *MED15* plasmids. Sequence-verified, double-stranded DNA fragments (gblocks) corresponding to two regions of the *MED15* gene with a restriction site substituted for each of the two major polyglutamine tracts, were synthesized by Integrated DNA Technology (IDT, Coralville, IA). Gblocks were individually ligated into the PCR 2.1 topo vector for storage and amplification: Q2:*Afe*I (pDC1012), Q3:*Bmg*BI (pDC1013), Q2:AfeI+Q3:BmgBI (pDC1014) and KIX (pDC1025).

Plasmids with synthetic *MED15* genes consisting of Q tracts from specific alcoholic beverage alleles and lacking all other polymorphisms were constructed by a series of gap repair transformations. Starting plasmid pRS315 M-WT (SV286) (Kim *et al.*, 2008) was digested with *BstAPI* to create a gap between the Q1 containing region (Q1r) and the MAD region. The gap was repaired using the Q2/Q3 gBlock digested out of pDC1014 to generate pDC2149 (12Q-0Q-0Q). pDC2149 was then digested at the *Bmg*BI site located within Q3, and gap repaired using Q3 PCR (primers F+1682 and R+2385) from WY7 (25Q) and PY23 (27Q) to generate pJF2164 (12Q:0Q:25Q) and pJF2165 (12Q:0Q:27Q). Next, pDC2164 and pDC2165 were digested with *Afe*I located at Q2 and gap repaired with Q2 tracts amplified from WY7, WY15, SY20, and PY23 (each one having a different Q2 pattern) with primers F+854 and R+1781 to generate pDC2201 (12Q:WY7Q:25Q), pDC2203 (12Q:WY15Q:25Q), pDC2205 (12Q:SY20Q:27Q), and pDC2207 (12Q:PY23Q:27Q). Finally, the alleles were fully reconstructed by adding back the KIX domain and Q1 tract. pJF2201, pJF2203, pJF2205 and pJF2207 were subjected to complete digestion with *Bam*HI and partial digestion with *Bsu*36I to remove the Q1 region. The gap was repaired using Q1 tracts amplified from WY7 (21Q) and WY15 (28Q) using primers F+334 and R+831 plus the KIX gBlock which was digested out of pDC1025 to generate plasmids pDC2209-2214. The final products (as well as many of the intermediates) were confirmed by sequencing.

(A)

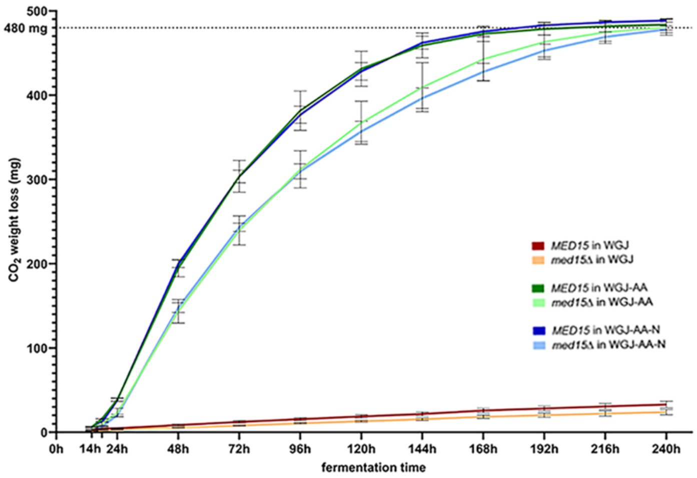

(B)

Equivalent Times to Weight Loss Benchmarks in WGJ-AA

Time (h)	N[1]	T10 (h)	T35 (h)	T50 (h)	T80 (h)
MED15	6	25.59	43.99	58.93	99.33
***med15*Δ**	6	29.63	54.84	73.66	133.8
**Adj. P-value**		3.12E-3	1.86E-3	2.65E-3	3.12E-3

[1]Biological replicates

(C)

Equivalent Times to Weight Loss Benchmarks in WGJ-AA-N

Time (h)	N[1]	T10 (h)	T35 (h)	T50 (h)	T80 (h)
MED15	6	26.98	45.068	59.48	99.839
***med15*Δ**	3	30.38	53.938	69.29	122.59
**Adj. P-value**		9.2 E-5	6.43E-3	2.66E-2	2.66E-2

[1]Biological replicates

(D)

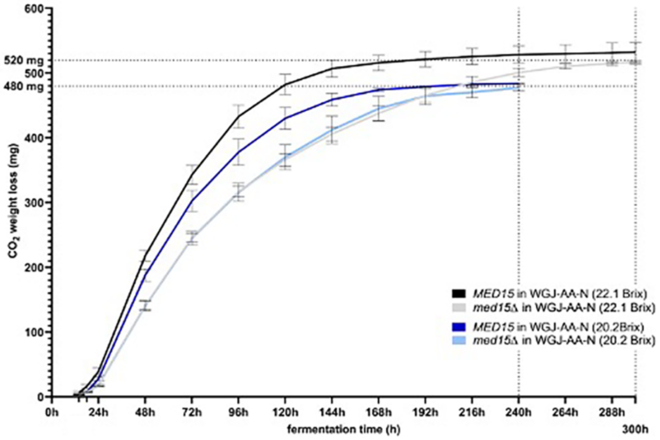

Figure S1. Increased yeast assimilable nitrogen (YAN) in the fermentation media had a minor effect on fermentation rate in the *med15*Δ mutant strain while media with a small increase in Bx affected CO_2 weight loss only in the wild type strain. **(A)** The impact of adding 1g/L $(NH_4)_2SO_4$ to elevate the YAN from 242.5 mg N/L to 434 mg N/L was evaluated. Minor differences in the time course, or in the time to fermentation benchmarks in the mutant strain were observed but the average maximum CO_2 weight loss was unchanged at 480 mg in nitrogen supplemented (WGJ-AA-N) or in regular (WGJ-AA) media. **(B-C)** Equivalent times to weight loss benchmarks in WGJ-AA **(B)** and WGJ-AA-N **(C)**. T10, 10%; T35, 35%; T50, 50%; T80, 80% of the maximum weight loss value (487 mg for WGJ-AA and 484 mg for WGJ-AA-N). **(D)** The impact of increasing the fermentable sugar from 20.2 to 22.1°Bx was evaluated. Weight loss was significantly higher at every time point in the higher Bx medium ($P_{adj}<0.01$) in the wild type strain with the average maximum CO_2 weight loss increasing from 483.8 to 528.4 mg, but was unchanged in the *med15Δ* mutant.

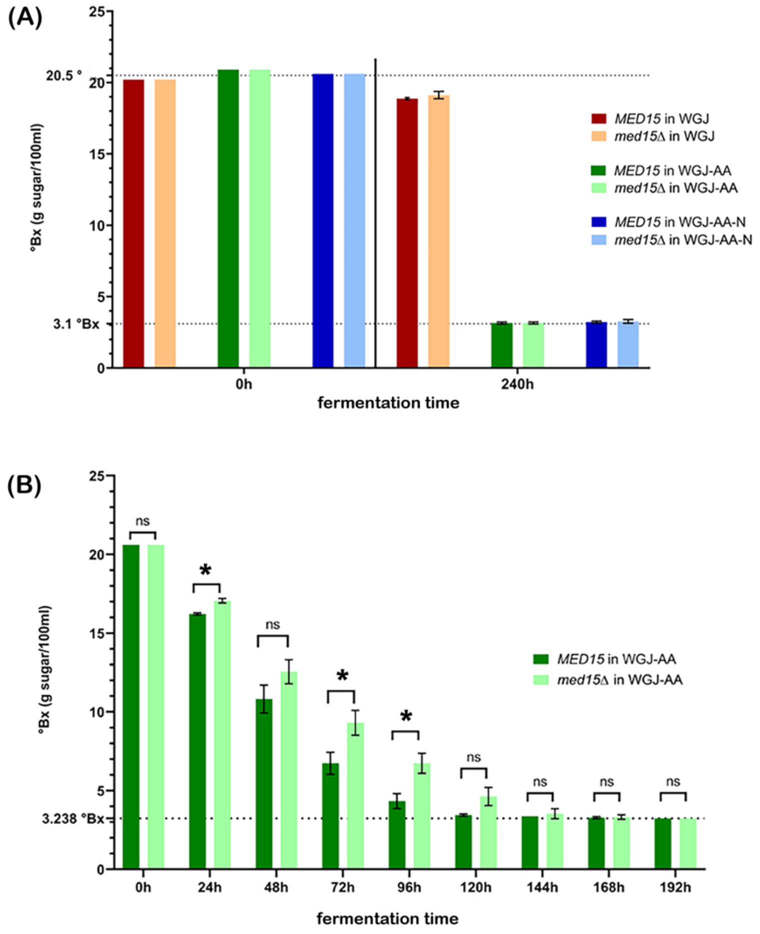

Figure S2. Sugar consumption is comparable but incomplete in both WGJ-AA and WGJ-AA-N media. Daily refractometer readings were used to assess residual sugar in vials inoculated and stoppered as for CO_2 loss measurements in WGJ-AA **(A)** or WGJ-AA-N **(B)** fermentations. 0.2 mL samples were taken from each vial by inserting a 1 mL syringe fitted with a 25G needle through the rubber membrane. Refractometer readings in °Bx were conducted in biological duplicate. Refraction due to accumulating ethanol was accounted for (Son *et al.*, 2009). Significance was determined using two-tailed t tests with multiple testing correction. *, $p \leq 0.05$; **, $p \leq 0.01$; ***, $p \leq 0.001$.

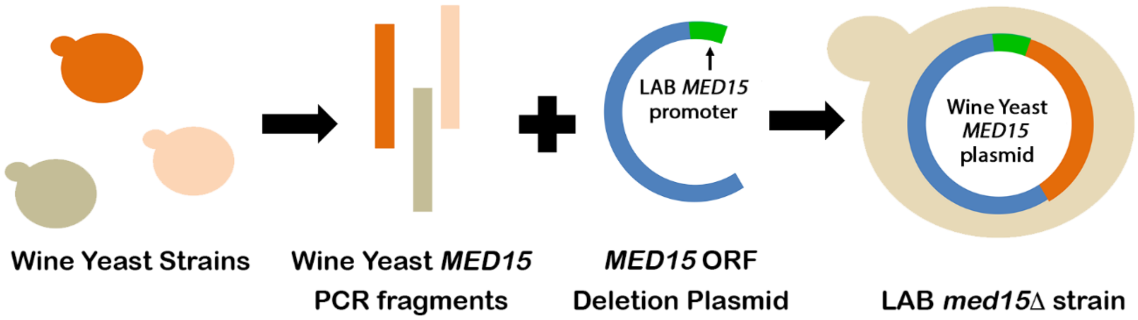

Figure S3. Construction of WY *MED15* expression plasmids. Plasmids were constructed by PCR amplification of the *MED15* gene from selected diploid wine yeast, recombined into a low copy (CEN) plasmid behind 0.7 kb of the LAB strain (S288C) *MED15* upstream regulatory sequences, and studied following transformation into *MED15*+ and *med15Δ* derivatives of S288C.

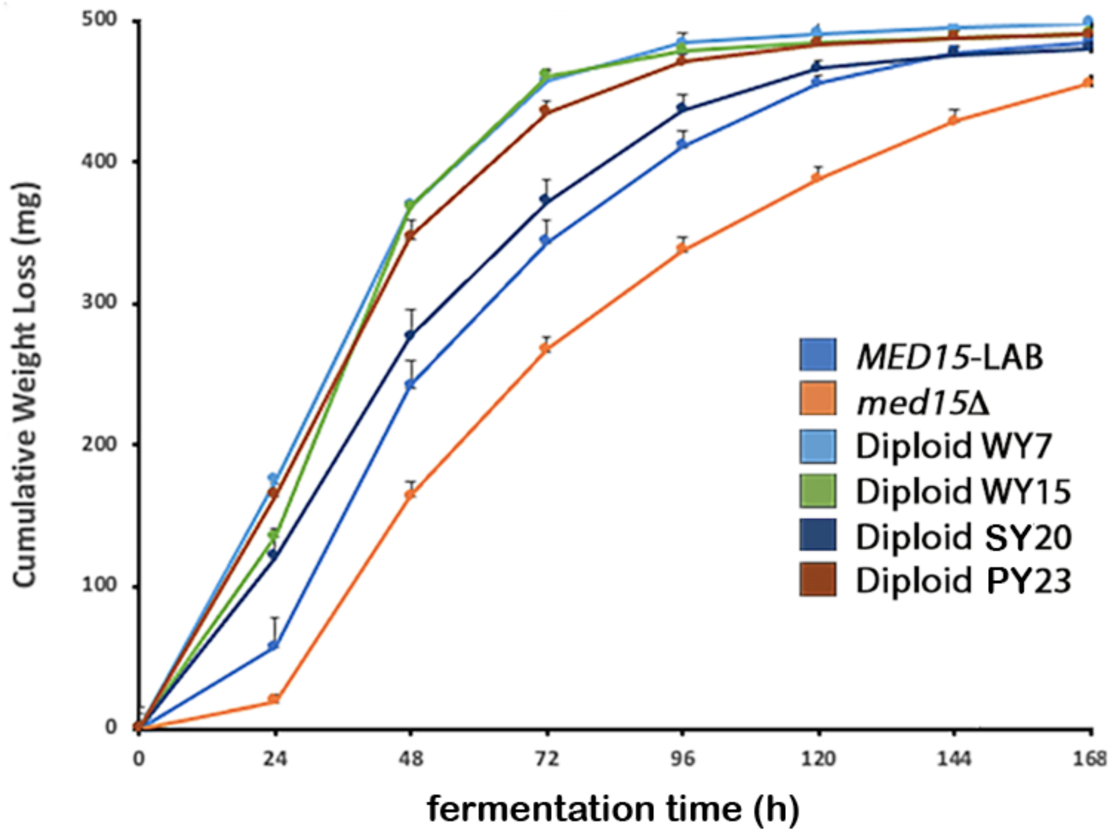

Figure S4. Comparison of the haploid WY *MED15* strains and the original diploid WY strains in fermentation. Fermentation curves for haploid strains with *MED15*-WY alleles were compared to original diploid wine yeast strains over 7 days. Data points are the averages of 3-8 biological replicates (transformants) for the LAB and *med15*Δ strains and two technical replicates for each diploid WY strain. Error bars are the standard deviation of the mean.

(A)

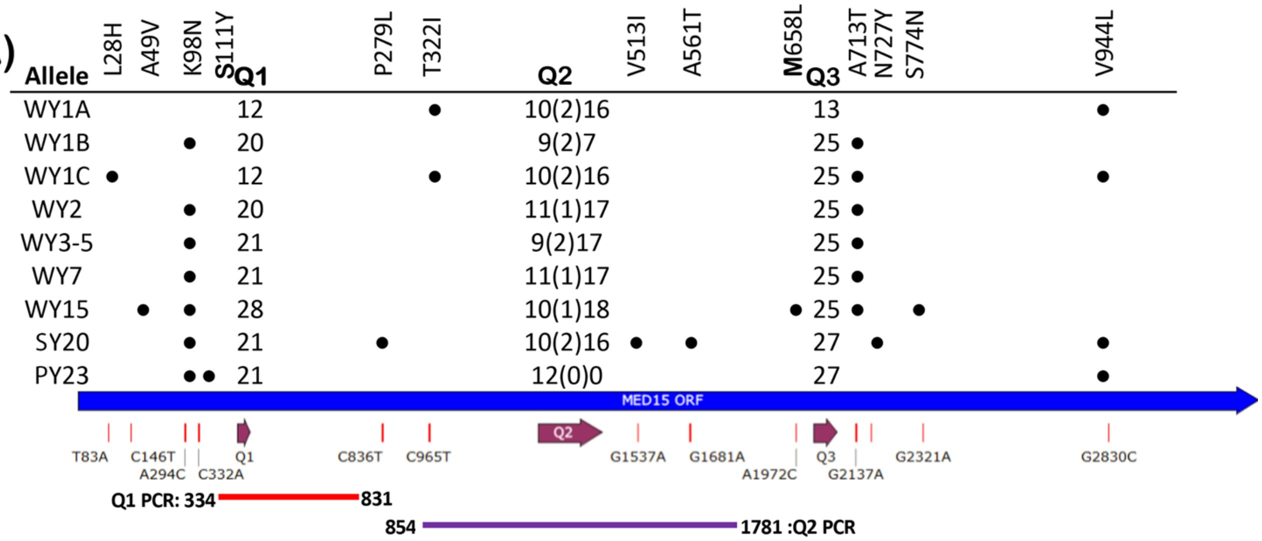

(B)

S288c			Allele											
nucleotide	SNP	amino acid	1.2	1.3	1.6	2.1	2.2	3	4	5	7	15	20	23
83	T->A	L28H			x		X	?						
146	C->T	A49V					X	?				x		
204	C->T	D68syn			x		X	?					x	x
294	A->C	K98N	x	x		x	X	?	x	x	x	x	x	x
300	G->A	V100syn					X	?						x
332	C->A	S111Y					X	?						x
	Q1													
486	G->A	L162syn					?							x
678	G->A	L226syn					?						x	
836	C->T	P279L					?						x	
965	C-> T	T322I	x		x		?							
1011	T->C	I337syn					?						x	x
1020	C->T	Y340syn					?						x	x
	Q2													
1537	G->A	V513I											x	
1681	G->A	A561T											x	
1965	G->A	Q655syn											x	
1972	A->C	M658L										x		
	Q3													
2109	T->G	T703syn		x	x	x	x	x	x	x	x	x		
2137	G->A	A713T		x	x	x	x	x	x	x	x	x		
2160	C->T	I720syn											x	
2179	A->T	N727Y											x	
2321	G->A	S774N										x		
2532	A->C	I844syn	x		x			?			?		x	x
2830	G->C	V944L	x		x			?			?		x	x
2922	T->C	F974syn	?		x			?			?			
3210	T->C	N1070syn	?	x	x	x	x	?	x	x	?			

Allele has SNP	x
Region not sequenced	?
Sequence from MiniGAL11	X
Nonsynonymous SNP	(shaded)

Figure S5. WY alleles differ from the LAB allele by the length of the Q tracts and by a small number of non-synonymous SNPs. (A) The Q tract sequence and the non-synonymous SNPS found in 11 different WY alleles are summarized. Below the table is shown a cartoon of the gene and each non-synonymous base pair change found among any of the WY strains. Non-synonymous

SNPs are shown in red, and synonymous SNPs are shown in gray. **(B)** A chart of synonymous (white) and non-synonymous (orange) SNPs present in each of the *MED15* alleles included in this study and their presence/absence in each of the 11 strains. Two synonymous SNPS (bp 2109 and bp 3210) and two non-synonymous SNPs (K98N and A713T) are shared by the most of the alleles characterized.

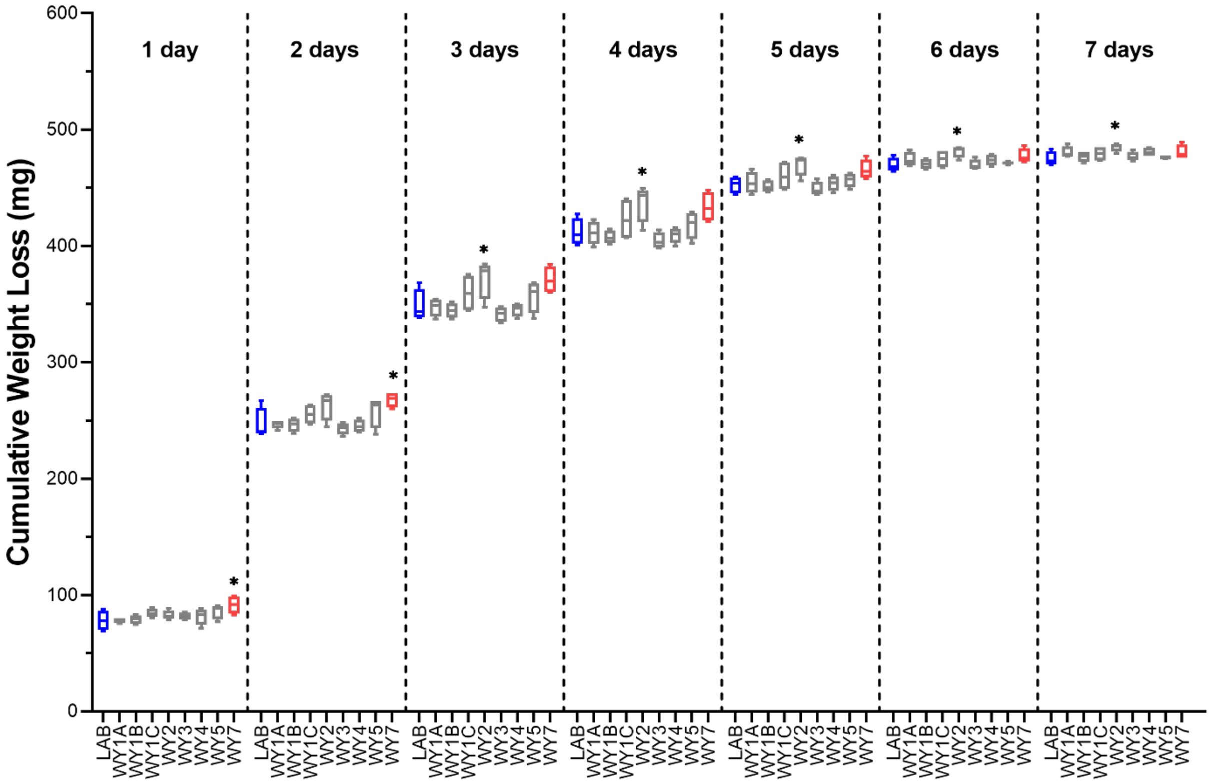

Figure S6. Additional European WY share fermentation kinetics with WY7. An 8-day fermentation profile (25°C) of strains carrying the *MED15* allele from additional European wine yeast alleles (A, B and C from WY1, WY2, WY3, WY4, WY5), compared to LAB, and WY7. Expected differences between LAB and WY7 are seen here, as well as differences between WY2 and WY7. Data points are the averages of 4 biological replicates (transformants), and error bars are the standard deviation of the mean. Significance was determined using ANOVA analysis with a Tukey post-hoc test. *, $p \leq 0.05$; **, $p \leq 0.01$; ***, $p \leq 0.001$.

(A)

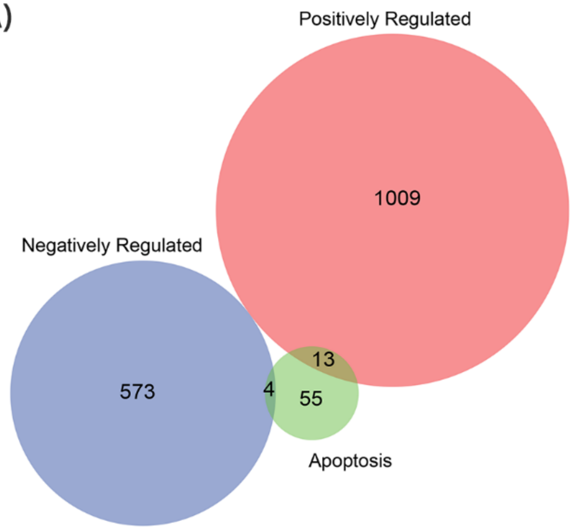

Apoptosis phenotype of null mutant
Increase
Decrease
Increase w/ overexpression

Standard Name	Log$_2$ fold change (Δ/*MED15*)	Gene Name
UBX4	-2.041	UBiquitin regulatory X
NDI1	-1.543	NADH Dehydrogenase Internal
HXK2	-1.469	HeXoKinase
RHO5	-1.466	Ras HOmolog
MSH2	-1.416	MutS Homolog
CLN3	-1.357	CycLiN
IPT1	-1.321	Inositol PhosphoTransferase
ESP1	-1.209	Extra Spindle Pole bodies
PAT1	-1.173	Protein Associated with Topoisomerase II
TPD3	-1.119	tRNA Processing Deficient
MAD1	-1.119	Mitotic Arrest-Deficient
WHI2	-1.114	WHIskey
DNM1	-1.106	DyNaMin Related
BMH1	1.065	Brain Modulosignalin Homolog
YLR162W	1.753	
OST2	2.057	OLigoSaccharylTransferase
SDD2	2.189	Suppressor of Degenerative Death

(B)

Biological Process Auto RIF - Predicted zscore	Number of Med15 regulated (pos) fermentation stress genes (44)	Adjusted P value
regulation_of_apoptotic_process_(GO:0042981)	13	6.5422E-14
glycogen_metabolic_process_(GO:0005977)	12	6.5422E-14
glycogen_biosynthetic_process_(GO:0005978)	12	6.5422E-14
regulation_of_actin_cytoskeleton_reorganization_(GO:2000249)	13	6.5671E-14
cellular_response_to_heat_(GO:0034605)	11	6.5671E-14
hyperosmotic_response_(GO:0006972)	11	8.3772E-14
ubiquitin-dependent_protein_catabolic_process_(GO:0006511)	12	1.867E-13
regulation_of_MAPK_cascade_(GO:0043408)	11	4.097E-13
regulation_of_endocytosis_(GO:0030100)	12	5.8594E-13
cellular_response_to_misfolded_protein_(GO:0071218)	12	8.9186E-13
regulation_of_cellular_localization_(GO:0060341)	12	8.9186E-13
misfolded_or_incompletely_synthesized_protein_catabolic_process_(GO:0006515)	11	8.9186E-13
response_to_ethanol_(GO:0045471)	10	4.4178E-12
**Phenotype Auto RIF - Predicted zscore**		
acquired_thermotolerance	11	6.67329E-11
lifespan	10	1.07061E-10
endocytosis	10	1.67213E-10
chronological_lifespan	9	2.43301E-10
nutrient_utilization	9	2.43301E-10
prion_loss	10	3.75668E-10
stress_resistance	8	1.18052E-09
flocculation	8	1.18052E-09
entry_into_G0_(stationary_phase)	8	1.32152E-09
heat_sensitivity	8	1.60079E-09

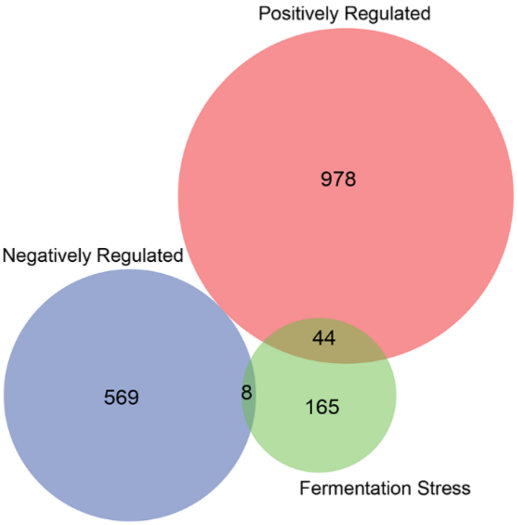

Figure S7. Subsets of genes regulated by *MED15* that may play a role in late fermentation. Intersections between genes that are differentially regulated by *MED15* (2-fold or greater) under normal growth conditions (Hu *et al.*, 2007) and genes annotated on SGD (https://www.yeastgenome.org/) as having a role in apoptosis **(A)** or genes differentially regulated in late fermentation (fermentation stress response (Marks et al., 2016) **(B)** are shown in proportional Venn diagrams and as gene lists (https://www.biovenn.nl/) (Hulsen *et al.*, 2008). Enrichment analysis for GO terms, phenotypes, and pathways was conducted using YeastEnrichr (https://amp.pharm.mssm.edu/YeastEnrichr/) for the genes regulated by both the fermentation stress response and by *MED15*.

Table S1. Plasmids used in this study

Plasmid	Details	Other comments[1]	Source or reference
**pRS315**	LEU2, CEN	SV31	(Sikorski and Hieter, 1989)
**pRS315 M-WT (SV286)**	Mini-*MED15;* the internal region of Med15 (aa 116 to 277) plus the Mediator association domain (aa 799 to 1081) located between the promoter and terminator regions of S288C *MED15* in pRS315 (LEU2, CEN)	Lab alias: SV286	(Kim *et al.*, 2008)
**pDC2141**	Lab *MED15* in pRS315 (LEU2, CEN)	SV286 gap repair	S288C allele
**pYJ2155**	WY7 *MED15* in pRS315 (LEU2, CEN)	SV286 gap repair	WY7:Table 6
**pYJ2156**	WY15 *MED15* in pRS315 (LEU2, CEN)	SV286 gap repair	WY15:Table 6
**pYJ2157**	SY20 *MED15* in pRS315 (LEU2, CEN)	SV286 gap repair	SY20:Table 6
**pYJ2158**	PY23 *MED15* in pRS315 (LEU2, CEN)	SV286 gap repair	PY23:Table 6

**pYJ2166**	WY1 *MED15* in pRS315 (LEU2, CEN)	Allele A, SV286 gap repair	WY1:Table 6
**pYJ2167**	WY1 *MED15* in pRS315 (LEU2, CEN)	Allele B, SV286 gap repair	WY1:Table 6
**pYJ2168**	WY1 *MED1* in pRS315 (LEU2, CEN)	Allele C SV286 gap repair	WY1:Table 6
**pYJ2169**	WY2 *MED15* in pRS315 (LEU2, CEN) in pRS315 (LEU2, CEN)	SV286 gap repair	WY2:Table 6
**pYJ2171**	WY3 *MED15* in pRS315 (LEU2, CEN)	SV286 gap repair	WY3:Table 6
**pYJ2172**	WY4 *MED15* in pRS315 (LEU2, CEN)	SV286 gap repair	WY4:Table 6
**pYJ2173**	WY5 *MED15* in pRS315 (LEU2, CEN)	SV286 gap repair	WY5:Table 6
**pDC1014**	Topo 2.1 with Q2:*Afe*I Q3:*Bmg*BI G block	(GC1014)	
**pDC1025**	Topo 2.1 with KIX G block	(GC1015)	
**pDC2209**	Reconstructed WY15 *MED15* Allele without SNPs in pRS315 (LEU2, CEN)		

**pDC2210**	Reconstructed SY20 *MED15* Allele without SNPs in pRS315 (LEU2, CEN)		
**pDC2212**	Reconstructed PY23 *MED15* Allele without SNPs in pRS315 (LEU2, CEN)		
**pDC2214**	Reconstructed WY7 *MED15* Allele without K98N SNP in pRS315 (LEU2, CEN)		

[1] Parenthetical names are lab storage designations

Table S2. Primers used in this study

Primer	Sequence (5’->3’)	Use
**MED15 F-245**	GGATGAGGATGATGAAGGTGC	Amplify MED15 ORF
**MED15 R+3498**	GTACTGATGATAGTCAAGTCCATTG	Amplify MED15 ORF
**MED15 F+384**	GTTCTTGAATCAGCAGGCTC	Screen WY alleles
**MED15 R+529**	GTGCCACTTTCATCTGGTTC	Screen & Sequencing
**MED15 F+1924**	GGCACTACTTCTACTGGAAAC	Screen WY alleles
**MED15 R+2143**	CATTAGCCATTGCCGAATAATTAGCCACACCAGG	Screen WY alleles
**MED15 F+334**	GCAACAGCGCCAATAATATGAATGTCAAT	Q1 PCR
**MED15 R+831**	GTTGTTCGCACTTGAATTGG	Q1 PCR
**MED15 F+854**	CACAAAATACCGTACCAAACGTCC	Q2 PCR
**MED15 R+1781**	CCCTCGGCACATTTTTCTAGGATC	Q2 PCR and Sequencing
**MED15 F+1682**	CCCCACAAGTCTACATCATCACAAAG	Q3 PCR
**MED15 R+2385**	CCTAGGAGTGGGTTGTTGACTAG	Q3 PCR and Sequencing
**MED15 F+334**	GCAACAGCGCCAATAATATGAATGTCAAT	Sequencing
**MED15 F+2693**	AGATGCTTACGTCATGCACTATCC	Sequencing
**MED15 R+2990**	GGGTTGCCGACATCCATATTAG	Sequencing

Supplementary Literature Cited

Birnboim, H.C., and Doly, J. (1979). A rapid alkaline extraction procedure for screening recombinant plasmid DNA. Nucleic Acids Res *7*, 1513-1523. doi: 10.1093/nar/7.6.1513

Chan, W.T., Verma, C.S., Lane, D.P., and Gan, S.K. (2013). A comparison and optimization of methods and factors affecting the transformation of *Escherichia coli*. Biosci Rep *33*. doi: 10.1042/BSR20130098

Ezeronye, O.U., and Legras, J.L. (2009). Genetic analysis of *Saccharomyces cerevisiae* strains isolated from palm wine in eastern Nigeria. Comparison with other African strains. J Appl Microbiol *106*, 1569-1578. doi: 10.1111/j.1365-2672.2008.04118.x

Guillaume, C., Delobel, P., Sablayrolles, J.M., and Blondin, B. (2007). Molecular basis of fructose utilization by the wine yeast *Saccharomyces cerevisiae*: a mutated *HXT3* allele enhances fructose fermentation. Appl Environ Microbiol *73*, 2432-2439. doi: 10.1128/AEM.02269-06

Hu, Z., Killion, P.J., and Iyer, V.R. (2007). Genetic reconstruction of a functional transcriptional regulatory network. Nat Genet *39*, 683-687. doi: 10.1038/ng2012

Hulsen, T., de Vlieg, J., and Alkema, W. (2008). BioVenn - a web application for the comparison and visualization of biological lists using area-proportional Venn diagrams. BMC Genomics *9*, 488. doi: 10.1186/1471-2164-9-488

Kim, D.H., Kim, G.S., Yun, C.H., and Lee, Y.C. (2008). Functional conservation of the glutamine-rich domains of yeast Gal11 and human SRC-1 in the transactivation of glucocorticoid receptor Tau 1 in *Saccharomyces cerevisiae*. Mol Cell Biol *28*, 913-925. doi: 10.1128/MCB.01140-07

Noguchi, C., Watanabe, D., Zhou, Y., Akao, T., and Shimoi, H. (2012). Association of constitutive hyperphosphorylation of Hsf1p with a defective ethanol stress response in *Saccharomyces cerevisiae* sake yeast strains. Appl Environ Microbiol *78*, 385-392. doi: 10.1128/AEM.06341-11

Sikorski, R.S., and Hieter, P. (1989). A system of shuttle vectors and yeast host strains designed for efficient manipulation of DNA in *Saccharomyces cerevisiae*. Genetics *122*, 19-27.

Son, H.S., Hong, Y.S., Park, W.M., Yu, M.A., and Lee, C.H. (2009). A novel approach for estimating sugar and alcohol concentrations in wines using refractometer and hydrometer. J Food Sci *74*, C106-111. doi: 10.1111/j.1750-3841.2008.01036.x

Urbanczyk, H., Noguchi, C., Wu, H., Watanabe, D., Akao, T., Takagi, H., and Shimoi, H. (2011). Sake yeast strains have difficulty in entering a quiescent state after cell growth cessation. J Biosci Bioeng *112*, 44-48. doi: 10.1016/j.jbiosc.2011.03.001

Watanabe, D., Araki, Y., Zhou, Y., Maeya, N., Akao, T., and Shimoi, H. (2012). A loss-of-function mutation in the PAS kinase Rim15p is related to defective quiescence entry and high fermentation

rates of *Saccharomyces cerevisiae* sake yeast strains. Appl Environ Microbiol *78*, 4008-4016. doi: 10.1128/AEM.00165-12

Watanabe, D., Wu, H., Noguchi, C., Zhou, Y., Akao, T., and Shimoi, H. (2011). Enhancement of the initial rate of ethanol fermentation due to dysfunction of yeast stress response components Msn2p and/or Msn4p. Appl Environ Microbiol *77*, 934-941. doi: 10.1128/AEM.01869-10

Zuchowska, M., Jaenicke, E., Konig, H., and Claus, H. (2015). Allelic variants of hexose transporter Hxt3p and hexokinases Hxk1p/Hxk2p in strains of *Saccharomyces cerevisiae* and interspecies hybrids. Yeast *32*, 657-669. doi: 10.1002/yea.3087
